# Evolution of TRIM5 and TRIM22 in Bats Reveals a Complex Duplication Process

**DOI:** 10.3390/v14020345

**Published:** 2022-02-08

**Authors:** Alexandre P. Fernandes, Ana Águeda-Pinto, Ana Pinheiro, Hugo Rebelo, Pedro J. Esteves

**Affiliations:** 1 CIBIO, Centro de Investigação em Biodiversidade e Recursos Genéticos, InBIO Laboratório Associado, Campus de Vairão, Universidade do Porto, 4485-661 Vila do Conde, Portugal; up201900698@edu.fc.up.pt (A.P.F.); anaagueda@cibio.up.pt (A.Á.-P.); ana.pinheiro@cibio.up.pt (A.P.); hugo.rebelo@cibio.up.pt (H.R.); 2 Departamento de Biologia, Faculdade de Ciências, Universidade do Porto, 4099-002 Porto, Portugal; 3 BIOPOLIS Program in Genomics, Biodiversity and Land Planning, CIBIO, Campus de Vairão, 4485-661 Vila do Conde, Portugal; 4 CIBIO/InBIO, Universidade de Lisboa, Tapada da Ajuda, 1349-017 Lisboa, Portugal; 5 CITS—Centro de Investigac¸ão em Tecnologias da Saúde, Instituto Politécnico de Saúde do Norte (IPSN), Cooperativa de Ensino Superior Politécnico e Universitário (CESPU), 4585-116 Gandra, Portugal

**Keywords:** Chiroptera order, TRIM proteins, restriction factors, gene duplication

## Abstract

The innate immunological response in mammals involves a diverse and complex network of many proteins. Over the last years, the tripartite motif-containing protein 5 (TRIM5) and 22 (TRIM22) have shown promise as restriction factors of a plethora of viruses that infect primates. Although there have been studies describing the evolution of these proteins in a wide range of mammals, no prior studies of the *TRIM6*/*34*/*5*/*22* gene cluster have been performed in the Chiroptera order. Here, we provide a detailed analysis of the evolution of this gene cluster in several bat genomes. Examination of different yangochiroptera and yinpterochiroptera bat species revealed a dynamic history of gene expansion occurring in *TRIM5* and *TRIM22* genes. Multiple copies of *TRIM5* were found in the genomes of several bats, demonstrating a very low degree of conservation in the synteny of this gene among species of the Chiroptera order. Our findings also reveal that *TRIM22* is often found duplicated in yangochiroptera bat species, an evolutionary phenomenon not yet observed in any other lineages of mammals. In total, we identified 31 TRIM5 and 19 TRIM22 amino acids to be evolving under positive selection, with most of the residues being placed in the PRYSPRY domain, known to be responsible for binding to the viral capsid during restriction in the primate orthologous TRIM proteins. Altogether, our results help to shed light on the distinctive role of bats in nature as reservoirs of viruses, many of which have become threatening zoonotic diseases through virus spillover in the last decades.

## 1. Introduction

Viruses play a unique role in the evolution of vertebrates, acting as a source of evolutionary pressure, and promoting events of fast coevolution [1]. As the host evolves mechanisms to restrict the virus activity, it becomes necessary for the virus species to find new ways to spread on, starting an often lengthy evolutionary arms race [2]. One of the ways species have been found to combat viruses is through restriction factors, which are important components of the innate host immune system with the ability to inhibit virus replication [3]. Some members of the tripartite motif-containing (TRIM) protein family are restriction factors that have previously been shown to have an important role in stopping viral infections in several mammals [4,5,6,7].

The human genome has approximately 100 genes coding for TRIM proteins [8,9]. These proteins are characterized by the presence of three canonical domains: an N-terminal RING domain, one or two B-Box domains, and a long coiled-coil domain, which together constitute the tripartite motif [10]. Many *TRIM* genes also code for a PRYSPRY (or B30.2) C-terminal domain, which contains four variable regions responsible for binding to viral exogenous material, making this domain crucial for viral restriction [11,12,13,14,15,16]. That is the case for the *TRIM5*, *TRIM22*, *TRIM6*, and *TRIM34* genes, which, in the human genome, are all clustered on chromosome 11, surrounded on both sides by olfactory receptors [17]. The study of TRIM5 has proven to be a source of fundamental information for the understanding of host–virus interactions. TRIM5ɑ was initially identified in cells of Old World monkeys as a blocking factor that binds to the capsid of the human immunodeficiency virus type 1 (HIV-1), protecting the host cells against cross-species transmission of retroviruses [7]. Although HIV-1 is shielded from restriction by the human TRIM5ɑ in most cell lines due to CypA–capsid interactions [18,19,20], mutations as small as two amino-acids can produce anti-HIV-1 activity on a similar level [21,22]. The specificity of TRIM5ɑ was previously assigned to its variable loops [11], which show strong evidence of positive selection, a phenomenon that reflects the coevolution between a restriction factor and its viral protein target. Several reports also suggest that TRIM22 is able to restrict the replication of different viruses, including encephalomyocarditis virus (ECMV), porcine reproductive and respiratory syndrome virus (PRRSV), influenza A, and HIV-1 [23,24,25]. TRIM22 has also been implicated in other biological processes, including cell differentiation and proliferation [26]. Though there is more research published about TRIM5 and TRIM22 and their roles in virus restriction, it has also been shown that TRIM34 and TRIM6 do play a role in the immune response; TRIM34 appears to be capable of restricting HIV in a TRIM5-dependent manner [27], whereas TRIM6 was shown to be important in IFN-I signaling pathways [28].

The origin of the *TRIM6*/*34*/*5*/*22* gene cluster happened in the ancestor of the Eutherians [26]. Evolutionary studies on this gene cluster have shown that it has had a dynamic evolutionary history, exhibiting strong signatures of positive selection and multiple events of gene expansion and gene loss [29,30,31,32,33]. For example, it was previously reported that rodents have an expanded paralogous cluster of at least eight *TRIM5*-*like* genes in mice, and three *TRIM5*-*like* genes in rats [29]. In the cow genome, an expanded clade of five *TRIM5* genes can be found, whereas there is no evidence for the presence of *TRIM22* [30]. In contrast, though *TRIM22* is functional in dogs, *TRIM5* is disrupted due to a frameshift mutation [29,30]. The dynamic evolution of the *TRIM6*/*34*/*5*/*22* cluster in other mammalian lineages, along with the importance of these proteins for viral cross-transmission defense, prompted us to investigate the molecular evolution of this cluster in bat species (order Chiroptera).

The Chiroptera order is one of the most diverse orders of mammals. The radiation of this order is believed to have happened around 60 million years ago (Mya) [34,35,36], and it can be divided into two suborders based on molecular genetics data, Yangochiroptera (composed exclusively of microbat families) and Yinpterochiroptera (composed by five microbat families and all megabat families). Bats are known to be reservoirs of viruses in the animal kingdom [37], and have been assigned as the source of many human diseases in modern times, including the recent outbreak of SARS-CoV-2 [38,39]. To our knowledge, no prior studies of the *TRIM6*/*34*/*5*/*22* gene cluster have yet been performed on bats, despite the fact Yinpterochiropteran bats have shown promising results in terms of viral restriction regarding the TRIM genes [4], increasing the group’s prominence for research in this field. Here, we provide the first detailed analysis of the evolution of the *TRIM6*/*34*/*5*/*22* in these mammals.

## 2. Materials and Methods

### 2.1. Phylogenetic Analyses

The coding sequences for *TRIM6*, *TRIM34*, *TRIM5*, and *TRIM22* were obtained from the NCBI database (http://www.ncbi.nlm.nih.gov, accessed on: 10 June 2021) using as reference *TRIM6*, *TRIM34*, *TRIM5*, and *TRIM22* coding sequences from *Homo sapiens* (accession numbers NM_001003818.3, NM_021616.6, NM_033034.3, and NM_006074.5, respectively). To better understand the evolution of these genes in the Chiroptera order, the genomes of seven Chiroptera families were analyzed (Vespertilionidae, Miniopteridae, Phyllostomidae, Hipposideridae, Pteropodidae, Rhinolophidae, and Molossidae), which resulted in a total of 113 sequences collected. In some species, assemblies on NCBI, the region containing the *TRIM6*/*34*/*5*/*22* cluster, was fragmented in multiple scaffolds. Because of this, it wasn’t possible to detect deletions that may have happened in the evolutionary history of bats, as there could be cases in which genomic information present in the actual chromosome was lost during sequencing. There were also cases in which collected sequences had to be removed because they contained premature stop codons or big insertions and deletions, which we considered to be either missannotations of pseudogenes or sequencing errors. Of a total of 113 sequences collected, 24 had to be removed from the dataset for such reasons, and will not be accounted for in the reported number of TRIM5 and TRIM22 paralogs in the following sections. Our database search resulted in a final dataset of 16 complete coding sequences for *TRIM6*, 16 for *TRIM34*, 31 for *TRIM5*, and 26 for *TRIM22*. The complete *TRIM* coding sequences were aligned using Clustal W2 (gap opening penalty of 10, and a gap extending penalty of 0.2) [40], followed by manual corrections when necessary. Amino acid alignments of TRIM6, TRIM34, TRIM5, and TRIM22 proteins were used to infer Maximum Likelihood (ML) phylogenetic trees with 1000 bootstrap repetitions using the MEGA-X software [41], with the substitution models JTT+G with four discrete categories determined using ProtTest [42].

A synteny map of the *TRIM6*/*34*/*5*/*22* gene cluster was then created for the species in which the whole gene cluster was contained in a single, unfragmented scaffold by tracking each collected sequence location using the NCBI Genome Data Viewer tool. For the synteny map, all genes annotated on NCBI were included, even those from which no good quality coding sequences could be extracted for the phylogenetic tree.

### 2.2. Molecular Evolutionary Analyses

To look for signatures of natural selection operating in TRIM5 and TRIM22, the HyPhy software implemented in the Datamonkey Web server [43], and codeml of the PAML v4.9 package [44] were used to detect codons under selection. When using the codeml of the PAML v4.9 package [44], a site-based model was used to determine if a model that allows positive selection (alternative model, M8) was a better fit to the data than a neutral model (null model, M7). The analysis ran with an initial ω ratio value of 1, and was conducted with the F3×4 model of codon frequencies. A Likelihood Ratio Test (LRT) was performed with two degrees of freedom to compare the fit of the two models by using the likelihood scores of the null neutral and alternative selection models. The Single Likelihood Ancestor Counting (SLAC) model, the Fixed Effect Likelihood (FEL) method [45], the Random Effect Likelihood, the Mixed Effects Model of Evolution (MEME) [46], and Fast Unbiased Bayesian Approximation (FUBAR) [47] methods were used. To avoid a high false positive rate, codons with *p*-values < 0.05 for SLAC, FEL, and MEME models, and a posterior probability > 0.95 for FUBAR were accepted as candidates for selection. For a more conservative approach, we used the approach described in previous studies, and only residues identified as being under positive selection in three or more ML methods were considered [32,48,49].

## 3. Results

### 3.1. Data Retrieving

**Figure 1:**
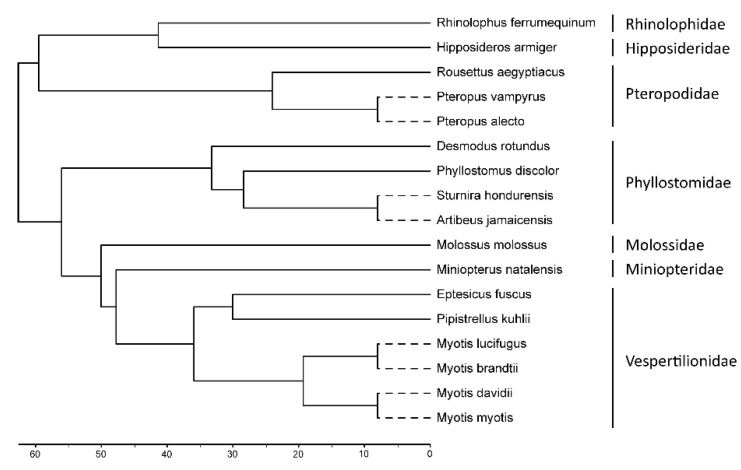
Phylogeny of the bat species included in this study. An ultrametric cladogram was generated using the iTOL tool [47], displaying updated phylogeny relations [35] of relevant members of the Chiroptera order. The length of branches represented by a dashed line is arbitrary and not proportional to the time scale.

To infer the evolutionary history of the *TRIM6*/*34*/*5*/*22* gene cluster in the Chiroptera order, we analyzed sequences available in the public NCBI database from 17 bat species distributed in 7 major families. In total, twelve genera from all the continents where bats are found are represented: *Myotis* (four species), *Pipistrellus* (one species), and *Eptesicus* (one species) from the family Vespertilionidae; *Miniopterus* (one species) from the family Miniopteridae; *Molossus* (one species) from the family Molossidae; *Sturnira* (one species), *Desmodus* (one species), *Phyllostomus* (one species), and *Artibeus* (one species) from the family Phyllostomidae; *Hipposideros* (one species) from the family Hipposideridae; *Rhinolophus* (one species) from the family Rhinolophidae; and *Pteropus* (two species) and *Rousettus* (one species) from the family Pteropodidae (Figure 1). At the end, a total of 89 TRIM genomic sequences (16 *TRIM6*, 16 *TRIM34*, 31 *TRIM5*, and 26 *TRIM22*) were retrieved among the different Chiroptera species (Table 1).

Among the different *TRIM* coding sequences obtained, detailed sequence analysis revealed a number of inconsistent annotations in the NCBI database. For example, *TRIM34* sequences from *P. vampyrus* (accession # XM_011383152.1) and *P. alecto* (accession # XM_015592331.2) presented a low degree of similarity when aligned with the remaining *TRIM34* sequences. In fact, after further analysis, which included manual and algorithmic comparisons with other *TRIM5* sequences, we determined that these were cases of missannotation. Both sequences were then correctly relabeled and included in the dataset. Moreover, we also found that a *TRIM6* transcript available for the *M. davidii TRIM6* loci (LOC102764759) was most likely a fused form between *TRIM6* and *TRIM34* transcripts. However, further investigation is required to determine whether a hybrid form of *TRIM6* and *TRIM34* is expressed in this species. For now, transcripts of both *TRIM6* and *TRIM34* were included in the dataset separately.

### 3.2. Phylogenetic Analysis Suggest Several Events of Duplication for TRIM5 and TRIM22 Genes

The final dataset of *TRIM* sequences from the different species of bats revealed that the analyzed *TRIM* genes present very different evolutionary histories across Chiroptera genomes (Table 1). In fact, though *TRIM6* and *TRIM34* genes have been preserved as a single copy in the analyzed bat species (Figure S1), *TRIM5* and *TRIM22* genes present a dynamic history of gene expansion in the studied bat genomes. This is especially remarkable in the case of *TRIM22*, which, as far as we know, was not yet found to be duplicated in any previous studies on other orders of mammals.

**Figure 2:**
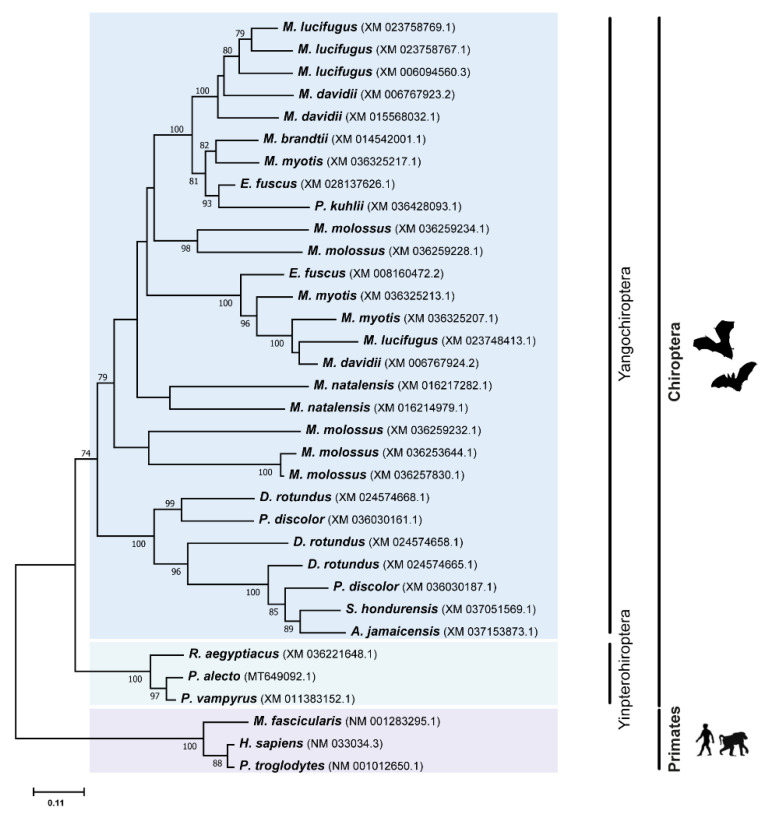
Phylogenetic analysis of TRIM5 protein in different Chiroptera species. The analyses were performed with 1000 generations and 1000 bootstrap searches. Bootstrap values (%) are indicated on the branches, and only bootstraps above 70% are shown.

In order to understand the relationships and evolutionary history of the different TRIM members, we performed a phylogenetic analysis for each one of these genes using a Maximum Likelihood (ML) method (for details, see Methods section). It was previously documented that the *TRIM5* gene was shaped by multiple duplication events during the evolution of mammals [17,29,33]. As can be seen from the TRIM5 ML tree (Figure 2), the obtained data confirms that the *TRIM5* gene has also undergone independent evolutionary expansions within different bat species. Several duplications were observed in the Yangochiroptera group, which led to some species accumulating up to five copies of *TRIM5* (Figure 2). Interestingly, no TRIM5 duplications seems to have occurred in Yinpterochiropteran bats, suggesting these events might be specific to bats from the Yangochiroptera group. Given the large number of duplications observed in some Yangochiropteran bats, and the fact that some basal branches in the ML tree display very low bootstrap values, it was not possible to precisely determine when all of the duplication events happened in this group. Nonetheless, it is clear that several duplication events happened at some point in different bat families, including in the Phyllostomidae, Molossidae, Miniopteridae, and Vespertilionidae families.

In the Phyllostomidae family, multiple copies of *TRIM5* can be found in species such as *P. discolor* and *D. rotundus*, respectively, with two and three copies, but not in *S. hondurensis* or *A. jamaicensis*, pointing towards a convoluted history of duplications and deletions of *TRIM5* in this group. In the Vespertilionoidea superfamily, a basal event of *TRIM5* paralogous expansion can be distinguished in the ML tree (Figure 2). This expansion in the copies of this gene affected all analyzed species of this superfamily, and, hence, it must have happened early in evolutionary history of bats, at around 50 million years ago [34,35,36]. In the *Myotis* genus, some recent duplications seem to have happened, which have led to a range of one to four *TRIM5* copies in the studied species of this genus. Some recent expansions of the *TRIM5* gene can also be observed in species of the Miniopteridae and Molossidae families, with both *M. natalensis* and *M. molossus* species presenting multiple *TRIM5* copies.

**Figure 3:**
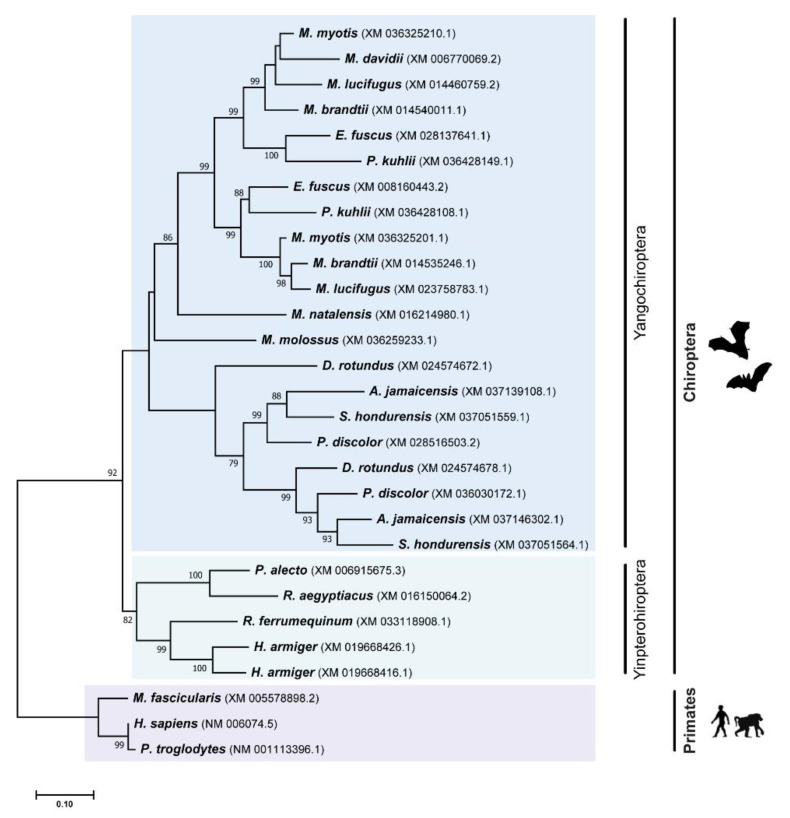
Phylogenetic analysis of TRIM22 protein in different Chiroptera species. The analyses were performed with 1000 generations and 1000 bootstrap searches. Bootstrap values (%) are indicated on the branches, and only bootstraps above 70% are shown.

Contrary to the dynamic evolutionary history of the *TRIM5* gene in mammals, different studies show that the *TRIM22* gene was always found to be either absent or present as a single copy in different mammalian genomes [17,29]. Unexpectedly, we found evidence that *TRIM22* has also been duplicated in the evolutionary history of bats (Figure 3). From the TRIM22 ML tree, two major clusters of bat species can be distinguished, supported by high bootstrap values (Figure 3). One of the clusters includes all TRIM22 amino sequences from Yinpterochiropteran bats, whereas the other includes all sequences from Yangochiropteran bats, matching the accepted Chiroptera phylogenetic relationships [35]. Only one duplication event was confirmed to have happened in Yinpterochiropteran bats, and it affected a single species, *H. armiger* (Figure 3). Comparing the two paralogous TRIM22 sequences from this species, we found out that they are very similar (94.9% identical), meaning that they must be product of a recent duplication. In Yangochiropteran bats, several events of gene duplication were observed. As can be seen in Figure 3, all Vespertilionidae species analyzed, except *M. davidii*, display two copies of *TRIM22*, which probably originated from a single duplication event that happened in a common ancestor at least 35.9 million years ago [35,50]. It was also possible to detect a duplication event that happened in an ancestor of the Phyllostomidae family. In this case, two copies of *TRIM22* were found in all species from the clade, putting its origin at around 33.23 million years ago [35].

**Figure 4:**
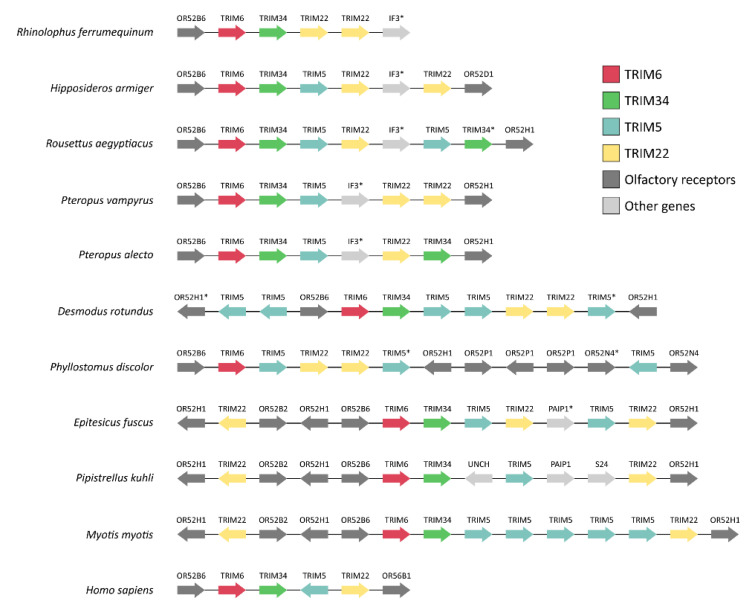
Synteny map of the *TRIM6*/*34*/*5*/*22* gene cluster of members of the Chiroptera order. Synteny comparisons of the *TRIM6*/*34*/*5*/*22* gene cluster of bats show very low conservation of this region of the genome among species. The human homologous gene cluster is shown at the bottom for comparison. Asterisks (*) indicate loci annotated as pseudogenes.

Next, the genomic organization of the *TRIM6*/*34*/*5*/*22* gene cluster and respective flanking genes from different bat species were analyzed (Figure 4). For that, a synteny map was made exclusively with bat species in which the *TRIM6*/*34*/*5*/*22* gene cluster was found in a single and unfragmented scaffold. The analysis of the flanking genes revealed a low conservation of the *TRIM6*/*34*/*5*/*22* gene cluster among the analyzed genomes, where differences in gene copy numbers and organization can be observed (Figure 4). However, the presence of a single *TRIM6* gene preceded by an olfactory receptor (Olfactory Receptor Family 52 Subfamily B member 6 (*OR52B6*) gene, or Olfactory Receptor Family 52 Subfamily H Member 1 (*OR52H1*) genes) is a common feature to all studied species. Overall, the genomic organization of the *TRIM6*/*34*/*5*/*22* gene cluster shows, once more, the presence of multiple copies of the *TRIM5* and/or *TRIM22* genes in different bat genomes, further supporting the hypothesis that multiple events of duplication are shaping the evolution of these genes.

### 3.3. Evidence of Positive Selection Acting on TRIM5 and TRIM22 Genes

**Figure 5:**
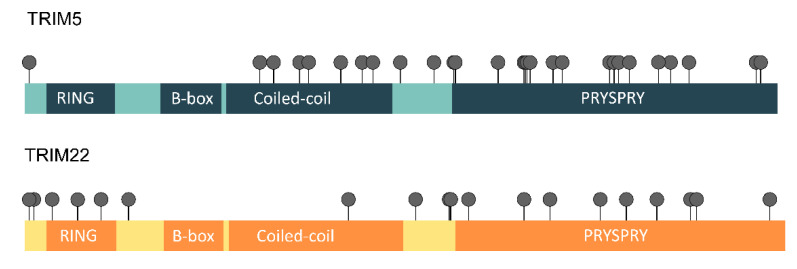
Amino acids under positive selection in TRIM5 and TRIM22. This diagram illustrates the position of the amino acids determined to be under positive selection in TRIM5 and TRIM22 by at least three of the five models of selection implemented (see Methods). In total, 31 TRIM5 positively-selected residues were detected, of which 7 were localized in the coiled-coil domain, 21 in the PRYSPRY domain, and 3 outside any domains. For TRIM22, of the 19 selected positions, 3 were found inside the RING domain, 1 in the coiled-coil domain, and 9 in the PRYSPRY domain. The remaining 6 residues are not placed within the bounds of any TRIM22 domain.

A common measure of natural selection acting on protein-coding genes is the ratio between nonsynonymous and synonymous mutations (dN/dS) [51]. Comparisons of the dN/dS ratios of primates have previously revealed that positive selection (dN/dS > 1) shaped the *TRIM5* and *TRIM22* genes in this taxon, particularly affecting the PRYSPRY and the coiled-coil domain of the analyzed sequences [17,31]. Posterior studies also found similar results when analyzing the *TRIM5* gene of rodents and lagomorphs [32,52]. To determine whether *TRIM5* and *TRIM22* have also evolved under positive selection in bats, we tested codons for selection under a variety of models implemented in the Datamonkey Web server and PAML (Table S1). To improve the quality of the data, only residues that were detected by three out of the five models implemented were considered to be under positive selection (see the Materials and Methods section). In total, 31 TRIM5 residues and 19 TRIM22 residues met this criterion (Figure 5). For TRIM5, the majority (21 out of 31) of the positively selected residues were localized within the PRYSPRY domain, frequently in one of its four variable regions (four in v1, six in v2, two in v3, and two in v4). Another seven residues were placed in the coiled-coil domain, whereas the remaining three were not within the limits of any domain.

## 4. Discussion

Although it has been demonstrated that many *TRIM* genes are capable of reducing the activity of retroviruses in some capacity, both *TRIM5* and *TRIM22* are unique in the sense that they interact directly with the virus to disable it. Studies on primates have demonstrated that this is done through the recognition of the viral capsid by these proteins’ PRYSPRY domain, which then binds the protein to the virus, and allows for restriction to take place [11,12,13,14,15,16]. However, because this process depends on the protein’s PRYSPRY domain being chemically compatible with antigens of the viral capsid, the fast-paced mutation rate characteristic of viruses ensues a strong selective pressure in the host protein, increasing the speed in which mutations are selected and fixed in this region of the gene. As a result, the PRYSPRY domains of TRIM5 and TRIM22 evolved quickly in bats, differing quite significantly from their counterparts in the human orthologous proteins (see Figure S2). It constitutes a classical, text-book example of the Red Queen hypothesis, with species competing and evolving in an endless “race”. This interpretation helps to explain the high inter-specific variation and number of positively-selected residues observed particularly in the PRYSPRY domains of TRIM5 and TRIM22, in contrast to the much more conserved TRIM6 and TRIM34, and has been previously evoked in the context of primate evolution [17]. Positively-selected residues such as these likely influence TRIM5 and TRIM22 binding to viral capsid proteins, which is reflected in little conservation among sequences from different species in these specific positions. Although in bats, no structural and functional studies have yet been performed to effectively determine the role of the PRYSPRY domain in TRIM5 and TRIM22 orthologues of this clade, the fact that the signature of positive selection of these genes is very similar to that seen in primates, with selected residues being mostly concentrated in the PRYSPRY domain and occasionally appearing in the coiled-coil domain [31], does suggest that both the domain and the proteins themselves may perform similar tasks in the cellular response to infectious hostile viruses in the Chiroptera order.

Because it is certain that more than one virus species is present amongst a population of mammals at any given time, it is tempting to speculate that the high amount of paralogous *TRIM5* genes observed in multiple groups of mammals has arisen to make it possible to fight all of the different varieties of viruses that affected them. Having multiple copies of *TRIM5* could be incredibly beneficial, as they would allow for independent mutations to appear in response to new viruses without compromising the efficacy of the protein in restricting the already known ones. Although this has been previously suggested as a reasonable explanation for the paralogous expansion of *TRIM5* in primates [17], it fails to provide an answer to why the same pattern had not yet been observed for *TRIM22*, which is functionally similar to the former. One possible reason for this is that the diversification of *TRIM5* was sufficient to counteract the variety of viral diseases in most mammals, and, therefore, the same process happening in *TRIM22* would be evolutionarily redundant. Another possibility would be that there is some undetermined difference in structure or function between *TRIM5* and *TRIM22* that makes the first prone to be duplicated, and the second not. Taking into account the fact that bats are known to serve as a reservoir of many virus species, one would be much more inclined to believe the first hypothesis to be correct, and speculate that the particular diversity of viruses in bats has led to the need for expansion of the *TRIM22* gene along *TRIM5*. It is possible, for example, that species of viruses better restricted by TRIM22 were abundantly present among bats in the past, and that the duplication of this gene is a result of this scenario. As for current known candidates for viruses which could be similar to those we hypothesize to have existed in bats, there are a few respiratory diseases, such as Influenza A and Porcine Reproductive and Respiratory Syndrome Virus (PRRS), as well as Encephalomyocarditis Virus (ECMV), all of which were shown to be inhibited by TRIM22, but not yet by TRIM5 [23,25]. Prominently, some varieties of Influenza A, such as H17N10, H18N11, and HL18NL11 [53,54,55], have been identified in Central and South American bats, perhaps hinting that the ancestors of these viruses were the ones driving duplication and positive selection of the *TRIM22* gene in bats. That said, no research has yet been performed to confirm links between the bat TRIM22 and the Influenza viruses mentioned, which are considerably different from the H1N1 and H3N2 strains commonly utilized in studies regarding TRIM22-mediated Influenza restriction [56].

## 5. Conclusions

This study of the evolution of the *TRIM6*/*34*/*5*/*22* gene cluster of bats reveals an unexpected evolutionary history in this order. The *TRIM5* gene was duplicated many times in the Chiroptera order, both recently and in ancestors of major bat families. Basal duplications were identified to have happened in ancestors of Vespertiolionidae and Phyllostomidae, whereas more recent duplications have multiplied the number of *TRIM5* copies in modern genera, leading to the accumulation of up to five paralogs in some species. Surprisingly, the *TRIM22* gene is also found duplicated in the Chiroptera order, a novel evolutionary phenomenon which had not yet been observed in any other mammals. Our results reveal that both of these genes have evolved under strong evolutionary pressures in Chiroptera, displaying high values of dN/dS ratios, and many positively-selected amino acids. Similarly to what was reported in primates, most of these selected positions are located inside the PRYSPRY domain, suggesting that the bat TRIM5 and TRIM22 likely perform analogous functions to their primate orthologues in restricting virus species. Altogether, these results are promising, and show that the TRIM22 has had a unique role in the evolution of bats, perhaps in restricting different varieties of viruses to those restricted by TRIM5. As for future work, we expect that the properties of TRIM5 and TRIM22 will be further investigated in order to examine the roles of these TRIM proteins in response to viral infection of bats or bat cells, since examining the interactions of these proteins with viruses could be of huge benefit to our understanding of evolutionary immunology.

## Figures and Tables

**Table 1:** Number of gene copies of *TRIM6*, *TRIM34*, *TRIM5*, and *TRIM22* found in different Chiroptera species.

**Group**	**Family**	**Species Name**	**TRIM6**	TRIM34	TRIM5	TRIM22
Yangochiroptera	Vespertilionidae	*Myotis brandtii*	XM_005860820.1	XM_005860818.2	XM_014542001.1	XM_014540011.1 XM_014535246.1
		*Myotis myotis*	XM_036325208.1	XM_036325206.1	XM_036325207.1 XM_036325213.1 XM_036325217.1	XM_036325201.1 XM_036325210.1
		*Myotis lucifugus*	XM_006094555.2	XM_023758764.1	XM_023758769.1 XM_023758767.1 XM_023748413.1	XM_014460759.2 XM_023758783.1
		*Myotis davidii*	XM_015560087.1	XM_015560147.1	XM_006767923.2 XM_015568032.1 XM_006767924.2	XM_006770069.2
		*Pipistrellus kuhlii*	XM_036428111.1	XM_036428091.1	XM_036428093.1	XM_036428149.1 XM_036428108.1
		*Eptesicus fuscus*	XM_008160441.2:134-1606	XM_008160439.2:174-1766	XM_028137626.1 XM_008160472.2	XM_028137641.1 XM_008160443.2
	Miniopteridae	*Miniopterus* *natalensis*	XM_016215031.1:626-2098	XM_016215034.1:166-1623	XM_016214979.1 XM_016217282.1	XM_016214980.1
	Molossidae	*Molossus molossus*	XM_036259561.1	XM_036258280.1	XM_036259234.1 XM_036259232.1 XM_036257830.1 XM_036253644.1 XM_036259228.1	XM_036259233.1
	Phyllostomidae	*Sturnira hondurensis*		XM_037037747.1	XM_037051569.1	XM_037051564.1 XM_037051559.1
		*Desmodus rotundus*	XM_024574677.1	XM_024574667.1	XM_024574668.1 XM_024574658.1	XM_024574672.1 XM_024574678.1
		*Phyllostomus* *discolor*	XM_028515503.2		XM_036030161.1 XM_036030187.1	XM_036030172.1 XM_028516503.2
		*Artibeus jamaicensis*	XM_037146300.1	XM_037146301.1	XM_037153873.1	XM_037146302.1 XM_037139108.1
Yinpterochiroptera	Hipposideridae	*Hipposideros armiger*	XM_019668446.1	XM_019668445.1		XM_019668416.1 XM_019668426.1
	Rhinolophidae	*Rhinolophus* *ferrumequinum*	XM_033120756.1	XM_033120754.1		XM_033118908.1
	Pteropodidae	*Pteropus alecto*	XM_006915678.1	XM_015592357.2	MT649092.1	XM_006915675.3
		*Pteropus vampyrus*	XM_011383131.1	XM_011383134.2	XM_011383152.1	
		*Rousettus* *aegyptiacus*	XM_016126836.2	XM_036221642.1	XM_036221648.1	XM_016150064.2

## Data Availability

Not applicable.

## Supplementary Materials

The following are available online at https://www.mdpi.com/article/10.3390/v14020345/s1. Figure S1: Phylogenetic analysis of (**A**) TRIM6 and (**B**) TRIM 22 proteins in different Chiroptera species. The analyses were performed with 1000 generations and 1000 bootstrap searches. Bootstrap values (%) are indicated on the branches, and only bootstraps above 70% are shown. Figure S2: Whole alignments used for the phylogenetic trees of (**A**) TRIM5 and (**B**) TRIM22. Table S1: Positive selection analyses for TRIM5 and TRIM22 of the order Chiroptera. (**A**) Amino acids under positive selection using five different methods; (**B**) TRIM5 alignment used to infer codons under positive selection; (**C**) TRIM22 alignment used to infer codons under positive selection.
