# Supplementary material for: Evolution of TRIM5 and TRIM22 in Bats Reveals a Complex Duplication Process
Source: Viruses. 2022 Feb 8;14(2):345. doi: 10.3390/v14020345 (PMC8879501; doi:10.3390/v14020345)
Supplement: Supplementary file 1 [file viruses-14-00345-s001.zip › Figure S1-╥╤╫¬╡╡.pdf]

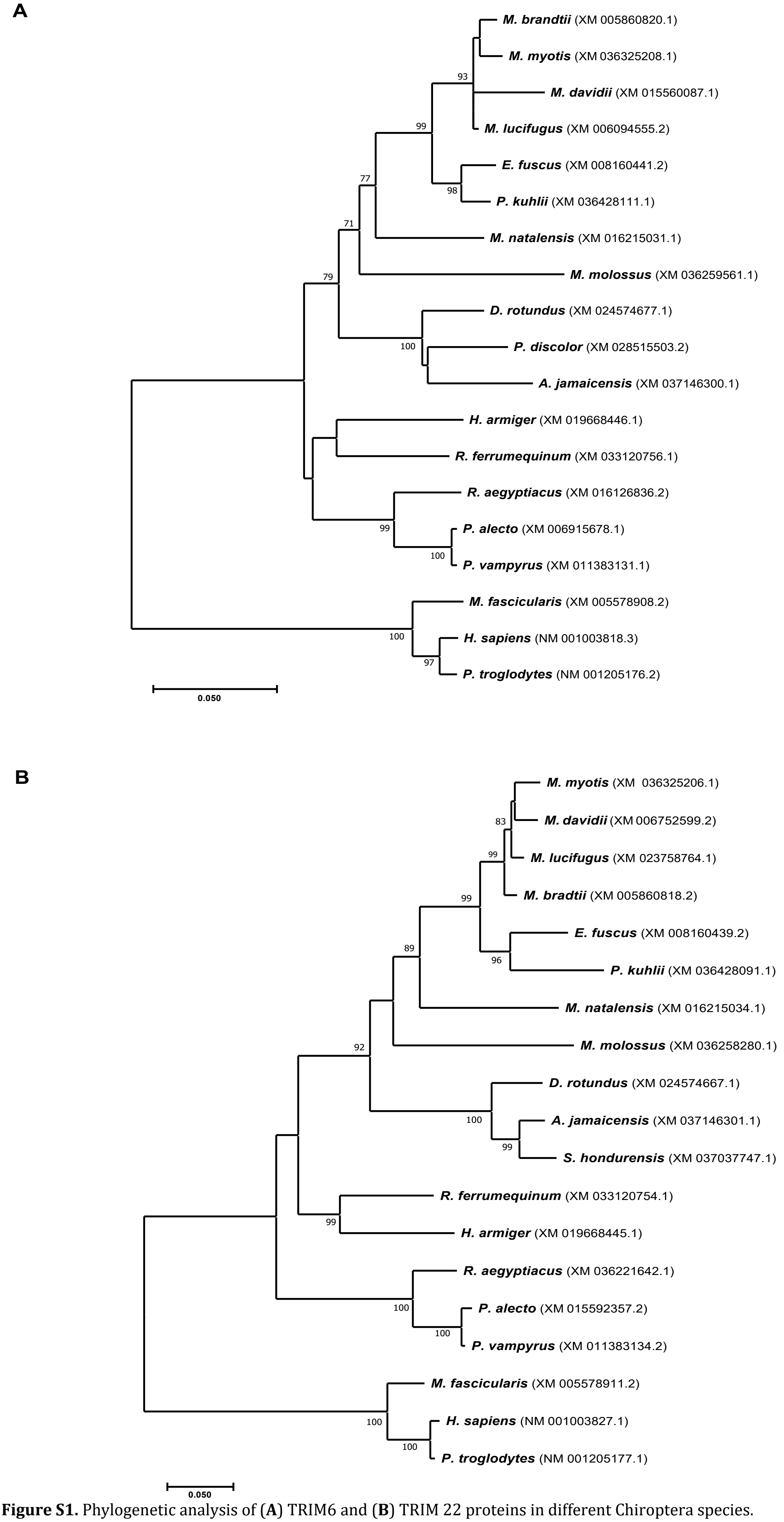

Figure S1. Phylogenetic analysis of (**A**) TRIM6 and (**B**) TRIM 22 proteins in different Chiroptera species.
