# Supplementary material for: Evolution of TRIM5 and TRIM22 in Bats Reveals a Complex Duplication Process
Source: Viruses. 2022 Feb 8;14(2):345. doi: 10.3390/v14020345 (PMC8879501; doi:10.3390/v14020345)
Supplement: Supplementary file 1 [file viruses-14-00345-s001.zip › Table S1.pdf]

Table S1. Positive selection analyses for TRIM5 and TRIM22 of Order Chiroptera.

A) Amino acids under Positive Selection using five different methods

Gene	Teste of Selection for PAML[c]					Amino acids under Positive Selection				Total of sites
	lnL[a] M7	lnL[a] M8	2Δ lnL[b]	p-value	Sites	SLAC[d]	FEL[e]	MEME[e]	FUBAR[f]	
**TRIM5**	-18295,7	-18105,8	379,7	<0.001	7, 80, 99, 165, 182, 188, 191, 198, 212, 284, 297, 302, 321, 324, 325, 328, 331, 333, 337, 338, 344-359, 370, 379, 393, 395, 396, 403, 404, 406, 408-413, 415-419, 426, 428-433, 439, 441, 456, 464, 480, 482, 484-487, 496, 502, 549	4,185,212,226,284,297,298,327,348,350,352,373,379,410,413,432,456,487,502,549	4,55,74,98,151,159,168,185,191,212,226,233,253,264,284,289,297,298,327,348-350,352,373,379,393,405,410,413,416,427,430,432,456,475,487,502,547,549,553	4,5,6,8,11,15,16,17,18,19,22,55,72,82,159,168,185,191,205,212, 226,233,253,255,256, 264,284, 297,298,308,316,327,330,332,334,336-338,345,348-350,352,354,370,373,379,394,405,410-419, 426,428,431,432,442,443, 456,462,470,475,487,525,538,545,547,549,555,557	4,159,168,185,191, 212,226,233,253,284,297,348-350,359,379,410,413,416,419,430,432, 456,487,502,547,549	**31**
**TRIM22**	-13024,2	-12969,2	110,1	<0.001	4, 7, 10, 19, 37, 47-51, 54, 55, 64, 69, 72, 81, 82, 84, 87-89, 91, 93, 94, 99, 104-106, 118, 128, 133, 134, 155, 177, 186, 201, 205, 216, 219, 220, 222, 226, 243, 246, 248, 250, 252, 256, 259, 261, 275, 278, 281, 284, 288, 289, 293, 314, 320, 322, 329, 331, 332, 381, 385, 398, 409, 431, 451, 457, 468	7,19,69,94,215,283, 352,409,431,453,457,505	4,7,19,51,69,128,215,261,283,284,332,338,352,382,385,399,409,431,453,475,505	4,7,13,19,29,37,42,51,52,69,70, 91,104-107,175,205,208,215,218,219,221,227,258,261,266,269,283,284,293,306,311,314,327,332,346,352,370,385,399,409,431,453, 455,465,505,508,	7,19,20,37,49,51,55,64,69,215,222,243, 256,259,261,284,288,293,332,352,385, 407,409,431,453,457	**19**

a) lnL: log-likelihood scores.

b) 2ΔlnL: likelihood ratio test (LRT) to detect positive selection.

c) Codons with posterior probabilities >90% in the BEB analyses (*: P>95%; **: P>99%).

d) Codons with significance level <0.1

e) Codons with significance level <0.05

f) Codons with posterior probabilities >0.90

B) TRIM5 alignment used to infer codons under positive selection

```
                                          10        20        30        40        50        60        70        80        90       100
                                 ....|....|....|....|....|....|....|....|....|....|....|....|....|....|....|....|....|....|....|....|
M. lucifugus (XM_023748413.1)    MASEILVNIKEMMTCPICLGILKEPRSLDCGHSFCQACITANNKESVNGQ-GESSCPVCRISYQPENLRPSLHLANIVEVLREVKLSPEEDQKSDLCVHH
M. lucifugus (XM_023758769.1)    MASGILVNIKEEVTCPICLELLTEPMSLDCGHTFCQACITTNNRASMIGQ--ESCCPVCRTSYQPENLRRNRHVANIVEALRKVKLSPQEEQKRDLCVRH
M. lucifugus (XM_023758767.1)    MASGILMNMKEEVTCPICLELLTEPMSLDCGHTFCQACITANNRGSIIGQ-GESCCPVCRISYQPENLRPNRHVANIVEALRKVKLSPKEEQKSDLCVHH
M. lucifugus (XM_006094560.3)    MASGILVNVKEEVTCPICLELHTEPMSLDCGHTFCQACITVNSRESMISQ-GESNCPVCRSTYQPENLRPNRYMANIVEALREVKLSPQEEQKRDLCVHH
M. molossus (XM_036253644.1)     MASRVLGNIKEEATCPICLELLKEPMSLDCGHSFCQDCITANNKEL--GQEGESHCPVCRLSYQPGNLRPSRHLANIVEMLRNVKLNPEEEQKRDLCVRH
M. molossus (XM_036259234.1)     MASGVLENLKEEVNCPICLELLKKPMCLDCGHSFCQACITANNKEPMTGQEGESHCPVCRLSYQPENLRPSRHLANIVETLREVKLSPEKKQKRDLCVHH
M. molossus (XM_036259232.1)     MASGILVNIKEEVSCPICLELLKEPLSLDCGHSFCQACITANNKESMMGQEGESHCPVCRLNYQPGNLRLNRHVANIVEMLREVRLIPEEEQKRNLCVRH
M. molossus (XM_036257830.1)     MASRVLGNIKEEVTCPICLELLKEPMSLDCGHSFCQACITANNKEL--GQEGESHCPVCRLSYQPGNLRPSRHLANIVETLRNVKLSPEEEQNRDLCVRH
M. molossus (XM_036259228.1)     MASGVLLNITEEATCPICLDLLKTPMCLDCGHSFCQACITTNNRLSMMGQEGESHCPVCRLSYQPGNLRPNVHLSNIVERLRKVKCSPEKDQKRDLCVRH
M. natalensis (XM_016217282.1)   MASEILVEIKEEVTCPICLELLTEPLSLDCGHSFCQACITTNSRQSMIRQEGESSCPVCRITYQPEKLRPNRHVASIVEALRKVKLNTEEELKRDLCERH
M. natalensis (XM_016214979.1)   MASEILEEIKEEVTCPICLELLTEPLSLGCGHSFCQACITTNSRQSMIRQEGESSCPVCRITYQPEKLQPSWKLSNIVETLQRVKLSTEEERKRDLCVHH
S. hondurensis (XM_037051569.1   MASGILVNMKEEVTCPICLELLTEPLSLDCGHTFCQACITANIKESIVS-GGESSCPVCRIRYQPGNLRPNRHVANIVEMLQKVKFSPEEEQKTELCERH
A. jamaicensis (XM_037153873.1   MASGILINMKEEVTCPICLELLTEPMSLDCGHTFCQTCISAN-KESMVS-EGESRCPVCRIRYQPGNLRPNRHVANIVEMLQKIKLSPEEEQKTDLCERR
M. brandtii (XM_014542001.1)     MASGILMNVKEEVTCPICLELLTEPMSLDCGHTFCQACITANNRESMIGQ-GESSCPVCRISYQPENLRPNRHVANIVEALREVKLSPQEEQKRDLCVHH
M. davidii (XM_006767923.2)      MASGIVVNMKKEVTCPICLELLTEPMSLDCGHTFCQACITAHNRESMICQ-RESSCPVCRSTYQPENMRPNRHVANIVEALREVKLSPQEEQKTDLCVRH
M. davidii (XM_015568032.1)      MASGILVNMKEEVTCPICLELLTEPMSLDCGHTFCQACITTNNRGSMIGQ-GKSSCPVCRITYQPENMRPNRHVANIVEALREVKLSLQEEQKRDLCVHH
M. davidii (XM_006767924.2)      MASGILGNIKEVMTCPICLEILTEPMSLDCGHSFCQACITANNKESVNGQ-GESSCPVCRISYQPENLRPSLHLANIVEVLREVKLSPEEEQKRDLCVHH
M. myotis (XM_036325207.1)       MASGILVNIKEVMTCPICMEILTEPMSLDCGHSFCQACITAKNKESVNGQ-GESSCPVCRISYQPENLRPSLHLANIVEVLREVKLSPEEEQKSDLCVHH
M. myotis (XM_036325213.1)       MASGILVNIKEVMTCPICLEILTEPMSLDCGHSFCQACITANNKESANGQ-GESSCPVCRISYQPENLRPSLHLANIVEVLREVKLSPEEEQKRDLCEHH
M. myotis (XM_036325217.1)       MASGILVNIKEEVTCPICLELLTEPMSLDCGHTFCQACITANNRDSMIGQ-GESSCPVCRITYQPENLRPNRHVANIVEVLREVKLSPQEEQRTDLCVRH
E. fuscus (XM_028137626.1)       MASGILVNIKEEVTCPICLELLTEPMSLDCGHTFCQACITANNRESMIGQ--DSSCPVCRISYQPENLRPNRHVANIVETLREVKLSPQEEQKRDLCVQH
E. fuscus (XM_008160472.2)       MASGVLMNIKEVVTCPICLEILTEPMSLDCGHSFCQACITANNKESMIS-EGESSCPVCRIRYQPENLRPSLHLANIVEVLREVKLSPEEEQKSDLCVHH
P. alecto (MT649092.1)           MATGILVNIQEEVTCPICLELLTEPLSLDCGHSFCQACITANSKEFVIGQ-GESSCPVCRVSYQPGNLRPNRHLANIAERLREVRLSPEEKWKRDLCVRH
P. alecto (XM_015592331.2)       -------------------------------------------------------------------------------------------------VRH
P. vampyrus (XM_011383152.1)     MAASGELSVMYVFTVLLEGQ-------------------------------GESSCPVCRVSYQPGNLRPNRHLANIAERLREVRLSPEEERKRDLCVHH
R. aegyptiacus (XM_036221648.1   MATGILVNIQEEVTCPICLELLTEPLSLDCGHSFCQTCITAHSKEFVIGQ-GVSSCPVCRVSYQPENLRPNVHLANIADQLREVRLSPEEEQKRDLCGYH
D. rotundus (XM_024574668.1)     MDSEILLKLKEEVTCPICLKLLTEPLSLCCGHTFCQACITANNKESTVSKERESSCPVCQSSYQPGILQPNQHVANIVETLREANLSPEEEQKTDLCERH
D. rotundus (XM_024574658.1)     MASGILTNMKEEVTCPICLELLTEPLSLDCGHSFCQACITANNQESMVNEERESSCPVCRISYQPGNLRPNRHVANIVEMLREVKWSPEEEQNTDLCVRH
D. rotundus (XM_024574665.1)     MALGMLMNMKEEVTCPICLELLTEPLSLDCGHTFCKACITANSKKSMVSEEGESSCPVCRIRYQPGNLRPNRHVANIVEMFQKVKLNPEEEQKTDLCERH
P. kuhlii (XM_036428093.1)       MASGIL-NIKEEVTCPICLELLTEPMSLDCGHTFCQVCITANNSESMMGQ--ESSCPMCRISYQPENLRPNRHVANIVEALRKVKLSPKEEQKRDLCVHH
P. discolor(XM_036030161.1)      MASGILVNIKEEVTCPICLELLREPLSLDCGHTFCQACITANDKESIVSEKGEGHCPVCRIYYQPWNLRPNRHVANIVEMLQKVKLSPEEEQKTDFCVHH
P. discolor(XM_036030187.1)      MASGILLNMKEEVTCPICLELLTEPLSLDCGHTFCQACITANNTESMVSEEGGSSCPVCRIRYQPGNLRPNRHVANIVEMLQNVKLSLEEEQKTDLCERH

                                         110       120       130       140       150       160       170       180       190       200
                                 ....|....|....|....|....|....|....|....|....|....|....|....|....|....|....|....|....|....|....|....|
M. lucifugus (XM_023748413.1)    GEKLLLFCQEDGKIICWLCE-RSQE-HRGHQTFLIEEITQECKEKLQKALDSLSAKQQEAEKLRADLEKETTSWKDLIENEIQSIQDYFKELRGILDSEE
M. lucifugus (XM_023758769.1)    GEKLLLFCQEHGKIICWLCE-RSQE-HRGHHTFLIEEIAQEYKEKLQAALDRLKAEQQKAEKLDVDLEEERTSKKDKIENDLKSVQDYFKQLRRILASEE
M. lucifugus (XM_023758767.1)    GEKLLLFCQEDEKIICWLCE-RSRD-HRGHHTFLMEEIAQEYKEKLQAALDRLKAEQQKAEKLDDDLEEERTSKKDQIEHERKSVLYYFRELRGILAREQ
M. lucifugus (XM_006094560.3)    GEKLQLFCQEDEKIICWLCE-RSQK-HRGHHTFLMEEIAQKYKEKLQAALDRLRAEQQKAEKLDNDLEEERISKKDKIENDLKSVQDYFKQLRDILAREE
M. molossus (XM_036253644.1)     GERLLLFCKEDEKIICWLCE-RSQE-HRGHHTFFMEEVAQEYKEKLQEDLKRLMSKEQIAEKWREDIREERTSWKDQIENENQCVQDCFKRLREILDNEE
M. molossus (XM_036259234.1)     GEKLLLFCKEDEKIICWVCE-RSQE-HRGHHTFLMEEVAQEYKEKLQEALKRLKAEQQKAEKLGTDLEEQRTTWKDLIDKDSQSVQDCFKNVRGILDSEE
M. molossus (XM_036259232.1)     GERLLLFCKEDGKIICWLCE-RSQE-HRGHHTFLMEEVAQEYKEKLQRALEKLRENKGNAEKWEADLKEERISCEDQIENESQHIQHFFKQLRGFLDIEE
M. molossus (XM_036257830.1)     GERLLLFCKEDEKIICWLCE-RSQE-HRGHHTFLMEEVAQEYKEKLQEDLKRLMSKEQIAEKWREDIREERTSWKDQIENENQCVQDCFKRLREILDNEE
M. molossus (XM_036259228.1)     GEKLLLFCKEEEKIICWLCE-RSQE-HRGHHTFLMEEVAQEYKEYLQEGLKSLKAEQQKAEQMGADLEKQRTSWRDLIENDRQSIQDSFKKLREFVDSEE
M. natalensis (XM_016217282.1)   GEKLLLFCKEDEKIICWLCE-RSQE-HRGHPTFLMEEVAQEYKENLQATVDRLRGEQQKAEEWGSDIREQRTSWKNKIQNGNKFVQYYYKQLRGILDSEE
M. natalensis (XM_016214979.1)   GEKLHFFCEEHGKLICFVCE-RSQE-HRGHKVLIKEEAAQHYKEKLQADLDRLRGEQKKAKKWGSDIRKESNFRQNQVQKERKRVQDYFEHLRGILDTEE
S. hondurensis (XM_037051569.1   GEKLLLFCKEDGKFICWLCE-RSQE-HRDHHTLLTEEVAQEYKERLQAALDRLRAEQQKVKEFGADIREERISWENQIKSEIQSAQDCFKKMRDILCSKE
A. jamaicensis (XM_037153873.1   GEKLLLFCKEDGKFICWLCE-RSQE-HRDHHTFLIEEVAREYKEKLQAALDRLRAEQQKVKKFGTDIREERTSWENQIKSEIQSAQDYFKKMRDVLYSKE
M. brandtii (XM_014542001.1)     GEKLLLFCQEDEKIICWLCE-RSPE-HRGHHTFLIEEIAQK-----------------------------------------------------------
M. davidii (XM_006767923.2)      GEKLRLFCQEDEKIICWLCE-RSQE-HRGHHTFLMEEIAQEYKEKLQAALDRLKAEQRKAEKLDNDLEEEGTSKKDQIENELKSFHNYFKQLRDIFTSVE
M. davidii (XM_015568032.1)      GEKLVLFCQEDEKIICWLCE-RSQE-HRGHHTFLMEEIAQEYKEKLQAALDRLRAEHQEGEKLDDNLEEERTSKKDQIEHELKSVQDYFKQLRGILASEE
M. davidii (XM_006767924.2)      GEKLLLFCQEDGKIICWLCE-RSQE-HRGHHTFLMEEIAQEYKEKLQKALGSLSAKQQEAEKLRADLEKETTSWKDLIENESQSIQDCFKELRGILDSEE
M. myotis (XM_036325207.1)       GEKLLLFCKEHGKIICWLCE-RSQE-HRGHHTFLMEEIAQKYQEKLQKALDSLSAKQQEAEKLRADLEKETTSWKDLIYNEIQSIQDYFKGLRGVLDGEE
M. myotis (XM_036325213.1)       GEKLLLFCKEHGKIICLFCE-RSQE-HRGHHTFLMEEIAQEYKEKLQKALDSLSAKQQEAEKLGVDLEKETTSWKDLIENESQSIQDYFKELRGILDREE
M. myotis (XM_036325217.1)       GEKLLLFCQEDKKIICWLCE-RSRE-HRGHHTFLMEEIAQEYKEKIQAALERLRAEQKEAEKLDDNLEEERTSKKDKIENDLKSVQDYFKQLRGILASEE
E. fuscus (XM_028137626.1)       GERLLLFCKEDEKIICWLCE-RSRE-HRGHQTFLMEEIAQEYKEKLQAALDRLRAEQQKAEKLEDDLEEERTSKKDQIENELKSVQDCFKHLRGILASEE
E. fuscus (XM_008160472.2)       GEKLLLFCQEHGKIICWLCE-RSQE-HRGHHTFLMEEIAQEYKEKLQKALDSLIAKQQKAEKLGADLEEETTSWKNLIENESQSIQDYFKELRGILDHEE
P. alecto (MT649092.1)           GEKLLLFCKEDGKFICWLCE-RSQE-HRGHHTFLVEEVAQEYQKTLQAALEKLRKEQQEAERLEADIREQRTSWKNQIQNERHSVHDGFKQLRGILDSEE
P. alecto (XM_015592331.2)       GEKLLLFCKEDGKFICWLCE-RSQE-HRGHHTFLVEEVAQEYQKTLQAALEKLRKEQQEAERLEADIREQRTSWKNQIQNERHSVHDGFKQLRGILDSEE
P. vampyrus (XM_011383152.1)     GEKLLLFCKEDGKFICWLCE-RSQK-HRGHHTFLVEEVAQEYQETLQAALEKLRKEQQEAEKLEADIREQRTSWKNQIQNERHSVHDGFKQLRGILDSEE
R. aegyptiacus (XM_036221648.1   GEKLLLFCKEDGKFICWLCE-RSQE-HRGHHAFLVEEVAQEYQETLESTLEKLRKEQQEAEKLEADIKEERTSWKNQIQNERHSVHDGFKQLRGILDSEE
D. rotundus (XM_024574668.1)     GEKLLLFCKEDGKFICWLCE-RSQE-HRGHHTFLMEEVAQEYQENLQAALDRLRAEHQKAEKLEADIKKERTSWKNLMQNERQIVQDCFKKLRVFLDTEE
D. rotundus (XM_024574658.1)     GEKLLLFCKEDGKFICWLCE-RSQE-HRGHHTFLMEEVAQKYQEKLHAILNGLRAELQEAEKLEADTREERTSWENQIQNESCSIRDCFKQLRGILDSKE
D. rotundus (XM_024574665.1)     GEKLLLFCKEDGKFICWLCE-RSQE-HRCHHTLLMEEVAQEYQEKLQAALDTLRAEQQKVKKFGADIREERTSWENQIKNEIRSAQDCFKKLRDILYSKE
P. kuhlii (XM_036428093.1)       GERLLLFCKEDEKIICWLCE-RSQE-HRGHQTFLVEEIAQEYKEKLQAALDRLRAEQQKAEKLEDDLAEERTFKMDQIENEMKSVQDSFEQLRGILAIEE
P. discolor(XM_036030161.1)      GEKLLLFCKEDGKFICWLCE-RSQE-HRGHHTFLMEDVAQEYQEKLQAALHRLRAEHQEAEKLEADIKEERTSWKNQIQKEIQSVQNFFKKLKGILDTEE
P. discolor(XM_036030187.1)      GEKLLLFCKEDGKFICWLCE-RSQE-HRDHHTLLTEEVAQEYKEKLQAALNRLRAEQQQVKKFGADIREERTSWENQIKSEVQSAQDCFKKLRDILYNKE

                                         210       220       230       240       250       260       270       280       290       300
                                 ....|....|....|....|....|....|....|....|....|....|....|....|....|....|....|....|....|....|....|....|
M. lucifugus (XM_023748413.1)    QKELQRLEKEERDVLSYLAQGGSELIQKGQLLRDLTWDL--QRRLQGSTAEMLQDVNGI---LE----RSKTFTLK--KLKTLPKKQRRVFQVPDLRGML
```

```
M. lucifugus (XM_023758769.1)    QKELQRLEKEAAENLDDLAQAESELAQQRKLLRDLNSDL--EHRLQGSTVEMLQDVKGI---ME----RSKTFTLK--KPKTLPMKQRRVLQAPDLGGML
M. lucifugus (XM_023758767.1)    QKELQRLEKEAAENHDDLVQAESELAQQKKLLRDLISDL--EHRLQGSAVEMLQDVKGI---ME----RSKTFTLK--KPKTLPMKQRRVFRAPDLGGML
M. lucifugus (XM_006094560.3)    QNELQRLEKEAAENLDDLAQAERKLAQQRKLLRDLISDL--EHRLQGSAVEMLQDVKGI---ME----RSKTFTLK--KPKTLPMKQRRVLQAPYLGGMQ
M. molossus (XM_036253644.1)     QKELQRLKKEDGDVLNDLAQDESKLGKQSQLLKVLILDP--EHRLQGSTLEMLQDVNSI---MK----RSKTFILR--RPKTLVKKQRSVFKGPDLTEVL
M. molossus (XM_036259234.1)     QKELNILKQEETDVLNDLAQTECELVQQSQLLEDLISDL--EHRLQGSTLEMMQDVHGI---ME----RSRTFSLKTLTPKTLSKEQRRVFQAPDWQQIL
M. molossus (XM_036259232.1)     LKEMEKLEKKKENILNDLAQSESELVKHSQLLRDFMSDL--EYRLQGSTVEMMQDVNGI---ME----RSETFILK--KPEALYEKQRSVFQAPDLREML
M. molossus (XM_036257830.1)     QKELQRLKKEDGDVLNDLAQDESKLVKQSQLLKVLILDL--EHRLQGSTLEMLQDVNSI---MK----RSKTFILR--RPKTLVKKQRSVFKGPDLTEVL
M. molossus (XM_036259228.1)     QKMLKMLKEEETDVLNDLAQTECELVQQSQLLKNLISDL--ENRLQGSTLEMMQDVNSI---MK----RIKTFTLM--KPKNVSKIQGRVFQAPDLNGML
M. natalensis (XM_016217282.1)   QKELQRLEKEEGDNLDDLAQAESELVQQGQLLTDLISDL--EHRLQGSTVEMLQDVKSI---IK----RSQTFILR--KPEALSKKQRTVIRVPDLK-ML
M. natalensis (XM_016214979.1)   QKELQKLEEEERDDLKDLAQAESELVQEGQLLTDLISDL--EHRLQGSTAEMLQDVNGI---IE----RSKTFTLK--KPKTLPKKQVRVFQDPYLSKVL
S. hondurensis (XM_037051569.1   QEEVGKLEKEKRDILHDLEQSESEQVQQSLLLRDLISEL--EHRLQGSTMKMLQDVNGI---LE----SSQTFTLK--RPKLLSKEQRKVFRVPDLNGML
A. jamaicensis (XM_037153873.1   QEEVQKLEKEKRDIFSDLEQSESEQVQQTLLLSDLISEL--EHRLQGSTMKMLQDVNGI---LE----RSQTFTLK--RPKIFSKEQRRVFQVPDLNGML
M. brandtii (XM_014542001.1)     ----------------------------------------------------NKDVKGT---VK----RSKTFTLK--KPKTLSRKLRKVFQAPDLRGIL
M. davidii (XM_006767923.2)      QKELQRLKKEVAENLNDLAQAESELAQQRNLLRDLISDL--EHQLQGSAVELLQDVKGI---ME----RSKTFTLK--KPKTLPMKQRRMFQAPDLRGVL
M. davidii (XM_015568032.1)      QKELQRLEKEAAENLDDLAQAESELAQQRKLLRDLISDL--EHRLQGSAVEMLQRQKFRGMWIYFVVSRSKTFTLK--KPKTLPMKQRRVLQAPDLGGML
M. davidii (XM_006767924.2)      QKELQRLEKEKRDVLSYLAQGDSELIQKSQLLRDLTWEL--QRRLQGSTAEMLQDVNGI---LE----RSNTFTLK--KLKTLPKKQRRVFQVPDLRGML
M. myotis (XM_036325207.1)       QKELQRLEKEEKDVLSYLAQGDSELIQQSQLLRDLTWDV--QHRLQGSTAEMLQDVNGI---LE----RSKTFTLK--KLKALPKKQRRVFQVPDLRGML
M. myotis (XM_036325213.1)       QKEMQKLEKEKRYILSYLAQGSSELIQQSQLLRDLTCDL--QRRLQGSTAEMLQDVNGI---LE----RSKTFTLK--KLETISKEHRRQFQLPDLRGML
M. myotis (XM_036325217.1)       QKEQQRLEKEAAQNLDDLAQAESELAQQKKLLRDLISNL--EHLLQGSTVELLQDVKDT---ME----RSKTFTVK--KPRTLPTKQRTVFQAPDLRGVL
E. fuscus (XM_028137626.1)       QKELQRLEKEAAENLDDLAQAERELAQQRKLLRDLISDL--EHRLQGSTVEMLQDVKGT---MK----RSKTFTLQ--KPKSLSTKQRIVFQAPDLRWML
E. fuscus (XM_008160472.2)       QKELQRLEEEESDVLSCLAQGGSELTKQSQLLRDLIWDL--QCRLQGSTAKMLQDVNGI---LQ----RSMTFTLK--KPETLSKKQRRVFQVPDLRGML
P. alecto (MT649092.1)           QRELQKLKKDEGEALHYLAEAENELVQQNQLVRVLISDL--ERRLKGSTMEMLQDVNGI---ME----RSKTFTLK--KPKTIPKKQRRMFQVPDLRGML
P. alecto (XM_015592331.2)       QRELQKLKKDEGEALHYLAEAENELVQQNQLVRVLISDL--ERRLKGSTMEMLQDVNGI---ME----RSKTFTLK--KPKTIPKKQRRMFQVPDLRGML
P. vampyrus (XM_011383152.1)     QRELQKLKKEEGEALHYLAEAENELVQQNQLVRVLISDL--ERRLKGSTMEMLQDVNGI---ME----RSKTFTLK--KPKTIPKKQRRMFQVPDLRGML
R. aegyptiacus (XM_036221648.1   QRELQKLKKEEGEALHYLAEAENELVQQKQLVKVLISDL--ERRLKGSKMEMLQDVNGI---ME----RSKTFTLK--KPKTIPNKQRRMFQVPDLRGML
D. rotundus (XM_024574668.1)     KKELQKLVKEERDILHDLEQSESELVQQSQVLRDLISEL--EHRLQGSMRRMLQEVNDI---ME----RSKTFTLK--KSKPVSTKQRRVFQAPDLSGML
D. rotundus (XM_024574658.1)     QKELQKLEKEKKNIFHVLEQSESEQAEQSQLLRDLISEL--EHRLQGSTRKMLQDVNGI---LE----RSHTFTLK--KPKTVSKKRRRVFQVPDLNGML
D. rotundus (XM_024574665.1)     QEEVQKLEKEKRDIFHVLEQSESELVQQSLLLRDLISEL--ELRLQGSTMKMLQDVNGI---LE----SSQTFTLK--RPKILSKEQRRVFRVPDLNGML
P. kuhlii (XM_036428093.1)       QKELQRLEKEAAENHEDLAQAERELVQQKKSLRDLISDL--EHRLQGSVVEMLQDVKDT---MK----RSETFSLQ--KPNTLSTKQRRVFQPLDLRGML
P. discolor(XM_036030161.1)      QKELQKLKKDEGDILHDLEQSESKLVKQSQVLRDLISEL--EHRLNGSMRKMLQDVNGI---ME----RSKTFTLK--KPKTVSKEQRRVFQAPDLSEML
P. discolor(XM_036030187.1)      QEEVRKLEKEKIYIFHVLEQSESEQVQQSLLLRDLISEL--ELRLQGSTMKMLQDVNGI---LE----SSQTFTLK--RPKILSKEQRRVFRVPDLNGML

                                          310       320       330       340       350       360       370       380       390       400
                                 ....|....|....|....|....|....|....|....|....|....|....|....|....|....|....|....|....|....|....|....|
M. lucifugus (XM_023748413.1)    QVFNELAVVRRYWANMTLNH---RSNVDISAHKRNGRG----DFYRRALCPPEREDY--------------VVLGFPSITSGKHYWEVDVPKNKDWILG
M. lucifugus (XM_023758769.1)    QVFNEFPDVRRYWVHVTLDPPTDKSYNVISEDRRQVR-------PV--PLLYILADK------------TNFGVLGSPSITSGKHYWEVDVSQKHAWIMG
M. lucifugus (XM_023758767.1)    QVFNELTDVRHYWVHVTLDPPTDKSYNVISEDRRQVR-------SL--PLLYIIADK------------TNFGVLGSPSITSGKHYWEVDVSQKCAWIMG
M. lucifugus (XM_006094560.3)    QMLNELTDVRRYWVHVTLDPPTDKSNIVISEDRRQVR-------P---RRWFILFHR------------INFGVLGSPSITSGKHYWEVDVSQKRAWIMG
M. molossus (XM_036253644.1)     QKFTGLTWMRHYWVHVTLNPPKKKSNVDISAGGKEVRC----VLRQNTNCASEKCEN------------EDYGILGSLVITSRKYYWEVDVSEKRAWALG
M. molossus (XM_036259234.1)     QVFNTH---------VTLEYPMAIGNTHISEDKKQVAA----HFRLTRGFLY-GNDI------------EDHGVLGSPLITSGKHYWEVDVSNKYSWSLG
M. molossus (XM_036259232.1)     QAFAELTAARCYWVYITLNLPKNNPDVAISEDRREVRV----------LHWNTNVHE------------ADYDVLGSPVITSGKHYWEVDVSDKHAWILG
M. molossus (XM_036257830.1)     QKFTGLTWMRHYWVHVTLNPPKKKSNVDISADGKEVRC----VLRQNTNCTSEKCEN------------EDYGILGSLVITSWKYYWEVDVSEKRAWALG
M. molossus (XM_036259228.1)     QVFHGLTDAQRYWVPVTLDPFLHKSNVIISKDQRQVRP----VFSSRTLIFHQRNRY------------EDYGVLGSPRITSGKHYWDVDVSDKYAWVLG
M. natalensis (XM_016217282.1)   QAFMELTDARQYWVHFTLSLLKNDPDVAISLDQRQVKV-----LRWSTTDTLPENDY------------QDCDVLGSRFITSGKHYWEIDVSEKRAWILG
M. natalensis (XM_016214979.1)   PAFNELTDARRYWVHVTLDPPTDKANVIISADWKQVRP----AFYLRTLYLYQKGDC------------EDYGVLGSRPMTSGKHYWEVDVSGKYAWVLG
S. hondurensis (XM_037051569.1   QVFDELTDVRRYWDHITLDPPKDRRNVAISADLKQVRY----EYHREPQVQHEISFILGVNQENHFHQESNNGVVGVPSITSGKHYWEVDVSNKRTWILG
A. jamaicensis (XM_037153873.1   QVFDELADVRRYWDHITLDPPTDRRNVAISADLRQVRY----EYHCNPQVQHDFSFI-LGNHENRCHQEGNNGVVGVPSITSGKHYWEVDVSNKRTWILG
M. brandtii (XM_014542001.1)     ETFNELADVRRSWVHVTLDPPTDKSDIIISADRRQVKS----AFYLRTHYLYQKDND------------EDDGVLGSPPITSGKHYWEVDVSNKYAWVLG
M. davidii (XM_006767923.2)      EAFTELTDVRRYWEHVTLDPLMNKSNVLISMYRKQVR-------PD--HFWLLRGDN------------MDFGVLGSPSITSGKHYWEVDVSQKRAWIMG
M. davidii (XM_015568032.1)      QVFNELTDVRRYWVHVTLDTPMDKSNIAISEDRRQVT-------PV--HFLFLRSND------------EDCGVLGSPSITSGKHYWEVDVSQKRDWIMG
M. davidii (XM_006767924.2)      KVFNELTDVRRYWANMTLNHQ---SNVDISAYKRKGRSDF----YKRAHCPPEREDY--------------VVLGFPPITSGKHYWEVDVPQCQNWILG
M. myotis (XM_036325207.1)       EVFNELTDVRRYWANMTLNHQ---SNVNSMGRKVRDDF---TDYYERAHCPPEREDY--------------GVLGFPSITSGKHYWEVDVPQCQNWILG
M. myotis (XM_036325213.1)       QVFNELTEVRRYWANVTLDHRRANSNIAISAYEREVRSDF-SDFFQRTHCPPEREDY------------EDYGVLGLPSITSGKHYWEVDVSQKHAWILG
M. myotis (XM_036325217.1)       EAFTELTDVRRSWVHVTLDPPTDKSDIVISADRRQVKP----AFYLRTHYLYQKGYY------------EDYGVLGSPPITSGKHYWEADVSDKYAWVLG
E. fuscus (XM_028137626.1)       QVFNELTDVRRYWVHVTLDPPTDKSNIVISADRRQVKP----AFYLRTLYLYQKGNY------------EDYGVLGSPPITSGKHYWEADVTDKYAWVLG
E. fuscus (XM_008160472.2)       EVFNELTEARRYWANVTLDHQRAKSNIAVSVYEREVRSDFFGDYYQKARDPYEREDY------------DDYGVLGLPSITSGKHYWEVDVSQESAWILG
P. alecto (MT649092.1)           QMFNELTDVRRYWVHVILDIPENNPDIAISADRRQVL-------VCKKKDTYWNYYC------------NDYSVLGSPIITSGKHYWEVDVSMKSDWILG
P. alecto (XM_015592331.2)       QMFNELTDVRRYWVHVILDIPENNPDIAISADRRQVLV-------CKKKATYWNYYC------------NDYSVLGSPMITSGKHYWEVDVSMKSDWILG
P. vampyrus (XM_011383152.1)     QMFNELTDVRRYWVHVILDIPENNPDIAISADRRQVL-------VCKKKDPYWNDNC------------NDYSVLGSPIITSGKHYWEVDVSMKSDWILG
R. aegyptiacus (XM_036221648.1   QVFNELTDVRRYWVHMILDLPENNPNVAISADRRQVRY----VCVCNKNSPYWNDNC------------NDYSVLGSPSITSGKHYWEVDVSMKSEWILG
D. rotundus (XM_024574668.1)     QVFNELTDVRRYWVLITLDPPKNKQNVAISADRRQVRY----EHHLDA--NKNAS-----------------AVLGSLVITSGKHYWEVDVSEKQSWILG
D. rotundus (XM_024574658.1)     QVFD---------VHITLEHSNYGQNVAISEDQRQVSY----KQPYNSDVRCGYRSKSYNR-----HQDNNSAVLGSPFITSGKHYWEVDVSQKDAWILG
D. rotundus (XM_024574665.1)     QVFDELTDVRRYWVHITLDPPKDRRNVAISADHRQVRY----EYHRDSQVQHEFFFGFDIN-----RQESDYGVVGVPSITSGKHYWEVDVSNKHTWILG
P. kuhlii (XM_036428093.1)       EPFKELTDVRCYWVNVTLDPPPDESHIIISADRRQVKP----AFYLKTCDLYPKGSY------------KDYGVLGSLPITSGKHYWEVDVSDEYDWALG
P. discolor(XM_036030161.1)      QAFNQLTDVRRYWVHITLQPSMDKQNVAISADRMQVRY----EHHLDA--NTKAS-----------------AVLGSLAITSGKHYWEVDVSEKQSWYLG
P. discolor(XM_036030187.1)      QVFDELTDVRRYWDHITLDPPKDRRNVAISADLRQVRY----EYHCDPQVQRGFPCNIGINQENRFHQASNYGVVGVPSITSGKHYWEVDVSDKRTWMLG

                                          410       420       430       440       450       460       470       480       490       500
                                 ....|....|....|....|....|....|....|....|....|....|....|....|....|....|....|....|....|....|....|....|
M. lucifugus (XM_023748413.1)    VCIRKYPDFTGIDFG------------GKNKPV-----SFQYQPQ----------NGYWVIGLQNYSEYQAFVNS----SYSNPSPL------TLFVTVP
M. lucifugus (XM_023758769.1)    VYEEKYPDSNRMDFIRQI---------KNSQPV-----CSIFQPK----------YGYWVIGLQNHSEYKAFVDS----DSSDPMTL------TLFLSVP
M. lucifugus (XM_023758767.1)    VYGEKYPDSNRMDFLRQI---------INSQPL-----CSIFQPQ----------YGYWVIGLQNHSEYKAFVDS----DFSNPKTL------TLFLSVP
M. lucifugus (XM_006094560.3)    VYGGKYPDSNRMDFIRQI---------KDSQSV-----CSIFQPK----------YGYWVIGLENHSEYKAFVDS----ASSDSRTV------TLFLSVP
M. molossus (XM_036253644.1)     VCVEKCPDFRPGFVR----------QSNNCQHI-----YSRCQPK----------CGYWVIGLENQCEYNAFEDG----FNFNPSKV------PLKLTVP
M. molossus (XM_036259234.1)     VYSGKRPDLNTREFVIQD---------DC-KHV-----CSRYQPK----------FGYWVIGLQNRFDYKAFVNS----ASSDASTL------TLFLTVH
M. molossus (XM_036259232.1)     VCCVKHNYFS-------------------FHQD-----YSRYQPR----------LGYWVIGLQNNSEYKAFLDL----SNYWGIFS------SLVLTVP
```

```
M. molossus (XM_036257830.1)     VCVEKCPDFR-PGFVRQS---------NNCQHI-----YSRCQPK----------CGYWVIGLENQCEYNAFEDG----FNFNPSKV------PLKLTVP
M. molossus (XM_036259228.1)     VYGDNSPDANMMGFVRQG---------NCYQHV-----CSLYQPK----------YGYWVIGLQNHSEYKAFVDS----ASSNASAL------TLSLTVP
M. natalensis (XM_016217282.1)   VYCAKRISFQQG--------------------------YSKYQPQ----------LGYWVIGLQNQFEYKAFVDS----ATYNSWPYNIPSPRTLTLTVP
M. natalensis (XM_016214979.1)   VCEENPPDSNRKDFVRRG---------NKRQHV-----CSRYQPK----------NGYWVIGLENHSNYKAFVDS----PSSDPSTL------TFTLTVP
S. hondurensis (XM_037051569.1   VCSQKSPTLNPLYELKPLSVG---RKDRMASCFTFGNSLIAPVPR---------DDGYWVIQLVNGCAYNALAKS----SFSGLFKV------TLSPTVP
A. jamaicensis (XM_037153873.1   VCSQKSPSLNLLCELNGMFVG---RKVQTSPLFTFGTLVTTPAPK---------GDGYWVIQLVNGCEYNALTKF----SFSDVFKV------TLNPTVP
M. brandtii (XM_014542001.1)     VYGEECLDSNMMDFVKQD---------KKHQHV-----WSQYQPK----------YGYCVIGLKNHSEYKAFVDI----ASSDSTTL------NLYLSVP
M. davidii (XM_006767923.2)      VYGENYPGSIKMDFINLV---------NNSQPV-----CSRFQPK----------YGYWVIGLPNHSEYKAFVDS----ASSDSTTL------TLYLSVP
M. davidii (XM_015568032.1)      VYGKSFPSFSLKEVQRES---------LNRKPF-----CSRFQPK----------YGYWVIGLENHSEYKIFVDS----AYADSTTL------TLYLSVP
M. davidii (XM_006767924.2)      VCSRRYPVSTGIDFGG-----------KN-QPV-----CSRYQPK----------NGYWVIGLQKYSQYQAFVDS----DSPNPSPL------TLFLTVP
M. myotis (XM_036325207.1)       VCSRRYPVSTGIDFWE-----------KN-QPV-----CCQYQPK----------NGYWVIALQNYYQYQAFVDS----ASSNPSPL------TLFLTVP
M. myotis (XM_036325213.1)       VCVGKYPVSIRMDFGKQI---------KN-QPV-----SYRYQPK----------NGYWVIGLQNYSEYKAFVNS----ASSKPSFL------TLFLTVP
M. myotis (XM_036325217.1)       MYCEKCPDSNMMDFVKQDKKHIIVKHGKKHLHV-----CSKYQPK----------YGYWVIGLQNHSEYKAFVDS----ASCDSTTL------TLYLGVP
E. fuscus (XM_028137626.1)       VYGVKCPDSNMMDFVKQG---------KKRQHV-----CSQYQPK----------YGYWVIGLQNHSEYKAFVDSD----SSDPKTV------TLFLSVP
E. fuscus (XM_008160472.2)       VCVGKYPSSIRVDFGKKG---------KNHQPV-----CSRYQPK----------NGYWVIGLQNYSEYQAFVDSDPISLSSNPSSL------TLFLTIP
P. alecto (MT649092.1)           VYGKKCPNSKLMGFIRQ---------SKSYQHD-----FLRYQPK----------YGYWVIGLQNQLEYNAFEDS----SSPNPLII------TLSLNVP
P. alecto (XM_015592331.2)       VYGKKCPNSKLMGF-----------IRQSKSYQHD---FLRYQPK----------YGYWVIGLQNQLEYNAFEDS----SSPDPLII------TLSLSVP
P. vampyrus (XM_011383152.1)     VYGKKSALSKLMGFI---------RQSKSYQHD-----FSRYQPK----------YGYWVIGLQNQLEYNAFEDS----SSPNPLII------TLSLSIP
R. aegyptiacus (XM_036221648.1   VYGKKCPDSKLMGLL---------RQGKSYQHG-----FSRYQPK----------YGYWVIGLQNQLEYNAFEDS----SSPNPLII------TLSLNVP
D. rotundus (XM_024574668.1)     VYGKKHSDT-SSDQP---------------------------------------RNHFWIVTLDNQFDYAAFGES----SSSNPLKL------TLNVTVP
D. rotundus (XM_024574658.1)     VCCQKFPSLSLFYDLSEVDDG---RKVTCFPPV-----NTRNN------------NGYWVIGSWKQFEYNAFAES----SSSNPLKL------TLYLTIP
D. rotundus (XM_024574665.1)     VCSQKVPNLNLLHEFNGVFVD---KKVGLNQQF-----FFNDQPK----IKVF-SGGYWVIQLVNGCEYNALVKS----SFSNLLKL------PLSLTVP
P. kuhlii (XM_036428093.1)       VC----TDFRVT-FV--------------CRNA-----YSQYQPK----------YGYWVIGFQNHSEYKAFVDS----DSIDPMTI------PLFPNVP
P. discolor(XM_036030161.1)      VYGEKDSDTSSDQP----------KR------------------------------CFWVIGLENQFKYNVFGEF----SSFDPLKL------TPSLTVP
P. discolor(XM_036030187.1)      VCSEKSPSLNLLHELNGMFVD---RKVQRYQTF-----NIGIPSTLTGPVTTRTGGGYWVIHLEERCIYNALAKS----SFCNLFKV------ALSLTVP

                                         510       520       530       540       550       560
                                 ....|....|....|....|....|....|....|....|....|....|....|....|....|...
M. lucifugus (XM_023748413.1)    PDRIGVFLDFAACTISFLNITNHGFLIYKFSSCRFYQEIYPYFNSMEYPGSLKLVSPSS---------
M. lucifugus (XM_023758769.1)    PRRVGVFLDYDTGTVSFFNITNNGLLIYKFSSCSFSQKMFPYFNPMKCPASMTLCSPGS---------
M. lucifugus (XM_023758767.1)    PRRVGVFLDYDAGTVSFFNVTNGGLLIYKFSSCSFSQKMFPYFNPMKCTAPMTLCSPSS---------
M. lucifugus (XM_006094560.3)    PCRVGVFLDYDAGTVSFFNVTNDRLLIYKFSSCSFSQKMFPYFNPMNCPVSMTLCSPSS---------
M. molossus (XM_036253644.1)     LHRVGIFLDYNAGTVSFLNVTNHGFLIYKFSSCSFSQKVFPYFNPMTCTGTLKLCSSRPSFPLAHPAK
M. molossus (XM_036259234.1)     PRRVGVFLDYSARTVSFCNVTNHGTLIYKFSSCSFPQEIFPYFNPMKRRRPLKLCSPSS---------
M. molossus (XM_036259232.1)     PRRVGVFLDYNAGTVSFFNVTNYGDLIYKFSSCCFSQEVYPFFNPLHCTGCMTLCSPSS---------
M. molossus (XM_036257830.1)     LHRVGIFLDYNAGTVSFLNVTNHGFLIYKFSSCSFSQKVFPYFNPMTCTGTLKLCSSRPSFPLAHPAK
M. molossus (XM_036259228.1)     PQRVGVFIDYNAGTVSFFNISNHGFLIYKFSSCSFPENIFPYFNPMKCVAPMTLCSPRS---------
M. natalensis (XM_016217282.1)   PRRVGVFLDYNAGTLSFLNVTNNGFLIYKFSACSFSGECFPYFNPMKCTGPMTLCSSSS---------
M. natalensis (XM_016214979.1)   PHRVGVFLEYDAGTVSFFNITNHGFLIYKFSACSFSGKMFPYFNPMKCTAPMTLCSPSS---------
S. hondurensis (XM_037051569.1   PRRVGVFLDYDARTVSFYNITNHGFLIYKFSSCSFSQEMYPYFCPMTCDVPMTLCSPNS---------
A. jamaicensis (XM_037153873.1   PHRVGVFLDYDACTVSFYNITNHGFLIYKFSSCSFSQEMYPYFSPMTCDVPMTLCPPSS---------
M. brandtii (XM_014542001.1)     PRRVGVFLDYDAGTVSFFNITNHGFLIYKFSSCSFSQKMFPYFNPMKCTAPMTLCSPGS---------
M. davidii (XM_006767923.2)      PCRVGVFLDYDAGTVSFFNVTNHGFLIYKFSSCSFSQKMFPYLNPMKCPAPMTLCSPGS---------
M. davidii (XM_015568032.1)      PRRVGVFLDYDAGTVSFFNVTNHGFLIYKFSSCSFSQKMFPYFNPMKCTAPMTLCSPGS---------
M. davidii (XM_006767924.2)      PRRIGVFLDYTASTISFLNITNHGFLIYKFSSCCFNQEIYPYFDSMEYPGSLKLCSPSS---------
M. myotis (XM_036325207.1)       PDRIGVFLDYTASTISFLNITNHGFLIYKFSSCCFNHEIYPYFNSMDYSGSLKLCSKSS---------
M. myotis (XM_036325213.1)       PRRIGVFLDYKACTISFLNITNHGFLIYKFSSCCFNQEIYPYFNSMKYSDSLKLCSPSS---------
M. myotis (XM_036325217.1)       PRRVGVFLDYDAGTVSFFNVTNHGLLIYKFTSCSFSQKMFPYFNPMKCTAPMTLCSPGS---------
E. fuscus (XM_028137626.1)       PRRVGVFLDYDVGTVSFFNVTTNGFLIYKFSSCSFSQKMFPYFNPMKCTAPMTLCSPGS---------
E. fuscus (XM_008160472.2)       PRRIGVFLDYNACTISFLNITNHGFLIYKFSSCCFNREIYPYFNPMKCSDSLKLCSPSS---------
P. alecto (MT649092.1)           PCRVGVFIGHNAGTVSFFNVTSHGFLIYKFSSCSFSQEIFPYFSPMKCAAPMALCSPNS---------
P. alecto (XM_015592331.2)       PCRVGVFIDHNAGIVSFFNVTSHGFLIYKFSSCSFSQEIFPYFSPMKCAAPMALCSPNS---------
P. vampyrus (XM_011383152.1)     PCRVGVFIDHNAGTVSFFNVTNHGFLIYKFSSCSFSQEIFPYFNPMKCAAPMTLCSPNS---------
R. aegyptiacus (XM_036221648.1   PSRVGVFIDYKAGTVSFFNVTSHGFLIYKFSSCSFSQEVFPYFNPMKCAAPMTLSSPNS---------
D. rotundus (XM_024574668.1)     PHRVGVFLDYDAGTVSFYNVTNHGFLIYKFSSCSFPQKIYPYFSPMTCTAPMTLCSPSS---------
D. rotundus (XM_024574658.1)     PRRVGIFLDYDARTVSFYNVTNHGFLIYKFSSCSFSQRIDPYFNPMTCTAPMTVCYPIS---------
D. rotundus (XM_024574665.1)     PCRVGVFLDYDARTVSFYNVTNHGFLIYKFSSCSFSQEMYPYFSPMTCTVPMTLCSPSS---------
P. kuhlii (XM_036428093.1)       PCRVGVFLDYDAGTVSFFNVTANGFLIYKFSSCSFSQKMFPYFSPMEGTAPMTLCSPGS---------
P. discolor(XM_036030161.1)      PRRVGVFLDYDACTVSFYNVTNHGFLIHTFSSCSFSQKVYPYFCPMTCTGPMTLCSPSS---------
P. discolor(XM_036030187.1)      PRRVGVFLDYDARTVSFYNVTNHGFLIYKFPSCSFSQEMYPYFCPMTCTVPMTLCSPSS---------
```

C) TRIM22 alignment used to infer codons under positive selection

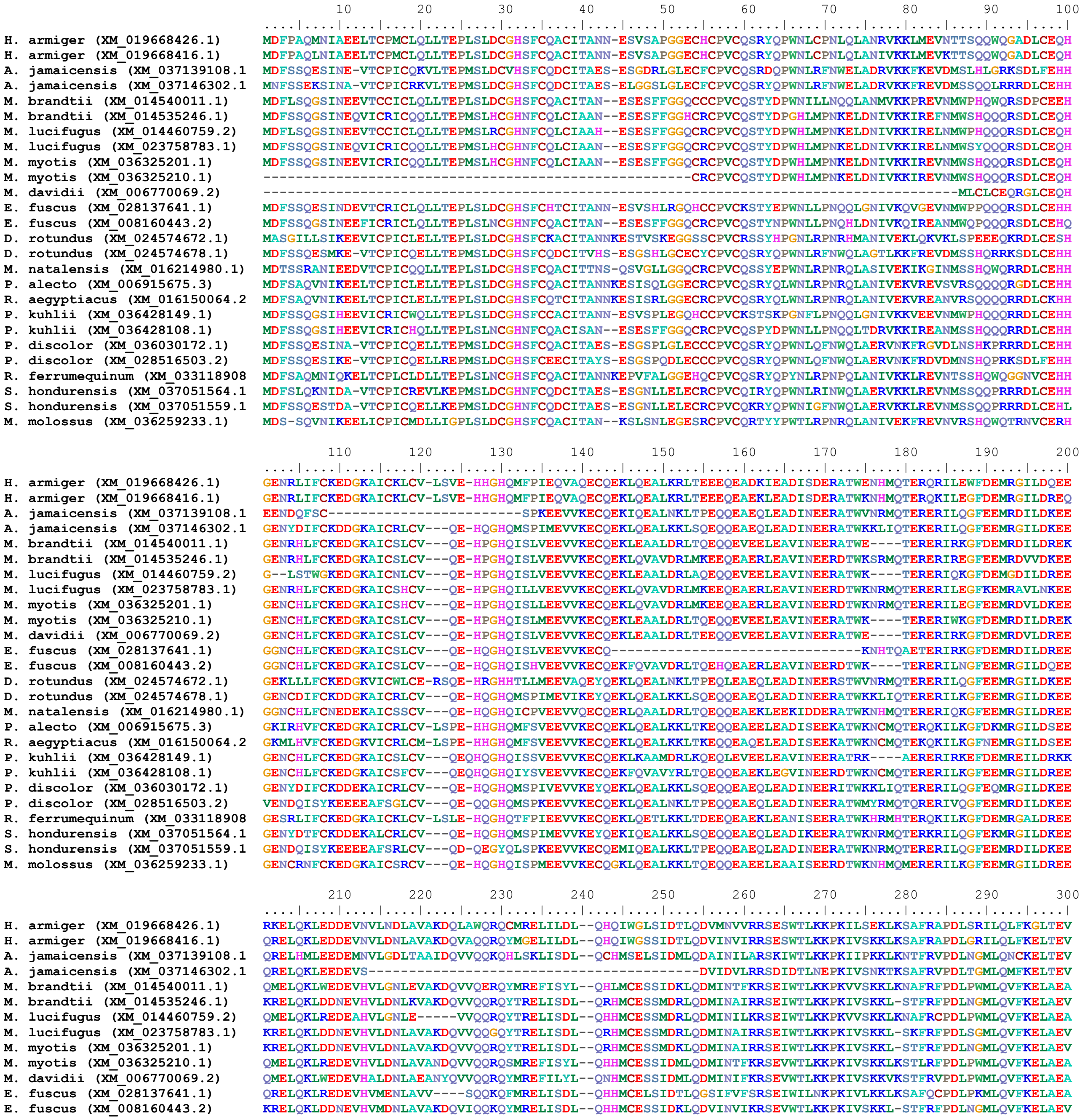

```
D. rotundus (XM_024574672.1)      QKELQKLEEDEVNVLGDLTAAIDQVVQQKQYLSELVTEL--QRHMCESSVDMLQDVINVLTRSKIWTLKKPKIIPKKLKSAFRVPDLNGMLQKCKELTEV
D. rotundus (XM_024574678.1)      QKELQKLEEDEVNV------------------SELHSDLQPHIPESIESIEMPQDVINILRRSEIDTLKKPKIVSNKAKSTFRVPDLNGMLQKFKELTEV
M. natalensis (XM_016214980.1)    RRELFQLEDDQVHVLDNLAMAKDQVAQQRQYMRELASGL--QCHNYESSVRTLPDMINIIRRSESWTLKKPKVFPKKLKSAFRLPDLSRMLQEFKELTGV
P. alecto (XM_006915675.3)        KKEMQKLEEVEVNVLDNLAVAKDQVAQQKQYMRELISDL--KCQMQGSSIDTLQDVINVIRRSESWTLKKPKIVPKKLKSTFRVPDLSGMLQGFKELTEV
R. aegyptiacus (XM_016150064.2    KKELQKLEENEVNVLDNLAVAKDQVAQQKQYMRELISDL--ERLMQGSSIDTLQDMINIIRRCESWTLKKPKIVSKKPKSAFRVPDLNGMLQGFKELTEV
P. kuhlii (XM_036428149.1)        HRELQKLREHEVHVLDNLAMVEDQVILKKQYTRELISDL--QRRMCELSIDTLQDMINTLKRNQIWDLSMPKIVLKKPKSAFRFPDLPHLLQVFKELAEA
P. kuhlii (XM_036428108.1)        KRELQKLDDNEVHVLDNLAVAKDQMAQQSQYMRELISDL--QRHMCEASVDTLQDIINVIRRSEVWTLKKPKIVSKKL-STFRFPDLNGMLKAFKELAEA
P. discolor (XM_036030172.1)      QRELQKLEEDEVNVNELTSDLQPHMPE------------------SVEAIDMPQDVINVLRRTEIDTLNKPKIVSNKAKSAFRFSDLTGMLQMFKELTEV
P. discolor (XM_028516503.2)      QRELQKLEEDEMNVLHDLTAAVDQVVQQKQYLSELVSDL--QYYMSESSVDTLQGVINVLTRSKIWTLKKPKIIPKKLKSAFQVPDLNGMLQKFKELTEI
R. ferrumequinum (XM_033118908    RKELQKLEEDEVNVLDNLAAAKDQVAQQRQYMSELISDL--QHQIRESTLDTLQDVINVIRRSESWTWKKPKIVSTKLKSAFQAPDLSRILQEFKELTEV
S. hondurensis (XM_037051564.1    QRELHKLEEDEVN-----------------------------------------DVINVLRRSEIDTLNKPKIVSNKTKSVFRVPDLAGMLQMFKELTEV
S. hondurensis (XM_037051559.1    QRELQKLEEDEMNVLDDLTAAIEQVVQQKQYLSQLVSDL--QCHVSELSIDMLQDAINILTRSKIWTLKKPKIIPKKLKSAFRVPDLHGMLQNCKELTEV
M. molossus (XM_036259233.1)      RKELYSLEEDEVNVLMNLATARDEVVQQRQYMRELISDL--QRHMCESSVDMLQDVINVIRRSEIWAFKKPKVVPRKLKTAFQVPDLSGMLQTFKELTEV

                                          310       320       330       340       350       360       370       380       390       400
                                  ....|....|....|....|....|....|....|....|....|....|....|....|....|....|....|....|....|....|....|....|
H. armiger (XM_019668426.1)       QSYWVDFMLNPVNALSGVVLSEDHRQVTVEKSFMFKNVYPCN---FSAFDVLGCQNFSSGKYYWEVDVSGKRAWILGVFSNTSNLNRKKSSGFVFDPNVS
H. armiger (XM_019668416.1)       QSYWVDFKLNPVNSPSAGLISEDQRQVTVEKSFMFKNVYPCN---PSSFDVLGCQNFSSGKYYWEVDVSGKRAWILGVFSNISNFNTKKSSGFVFNPNVN
A. jamaicensis (XM_037139108.1    QSYWVDLMLNPLNNLSNIAVSSDQRQVTFGQYSMFSNVYPCN---FSAFDVLGSRNFFSGKYYWEVDVSGKIAWLLGVYSTPSNLNRKKSSGFVFNPNVN
A. jamaicensis (XM_037146302.1    QSYWVDLMLNPLNAVSNVVISTDQRQVTVGQQSVFSDVHPCNPCDFSAFDVVGTQHFSSGKYYWEVDVSGKIAWLLGVYSAPSNLNRKKSSGFVFNPSVN
M. brandtii (XM_014540011.1)      QSYWVDLMLNPLNTLSDIIISEDRRQVTVGNYFMFWNVYPRD---FSALDVLGSQNFSSGKYYWEVDVSKKIAWILGVYSKTSNLNRKKSSGFVFNPDVN
M. brandtii (XM_014535246.1)      QNYWVDLTLNPFNDVSNVVVSEDQRQVTVGNDIMFRNVYPCN---FYAFDVWGSQNFSSGKCYWEVDVSGKIAWILGVYSKTSNLNRKKSSGFVFNPQTN
M. lucifugus (XM_014460759.2)     QSYWVNLMFNPLNTLSDIIISEDRRQVTVGNYFMFWNVYPHD---FSALDVLGSQNFSSGKYYWEVDVSKKIAWILGVYSKTSNLNRKKSSGFVFNPDVN
M. lucifugus (XM_023758783.1)     QNYWVDLTLNPFNDVSNVVVSEDQRQVTVGN-IMFRNVYPCN---FSAFDVWGSQNFSSGKCYWEVDVSGKIAWILGVYSKTSNLNRKESSGFVFKPQTN
M. myotis (XM_036325201.1)        QSYWVDLTLNPFNDVSNVVFSEDQRQVTVGNDIMFRNVYPCN---FSAFDVWGSQNFSSGKCYWEVDVSGKIAWILGVYSKTSNLNRKKSSGFVFNPQTN
M. myotis (XM_036325210.1)        QSYWVDLMLNPLNTLSDIIISEDRRQVTVGNYFMFWNVYPCD---FSALDVLGSQNFSSGKYYWEVDVSKKIAWILGVYSKTSNLNRKKTSGFVFNPDVN
M. davidii (XM_006770069.2)       QSYWGK-KLQVLQIAMDIIISEDRKQVTVGNYFMFWNVYPCD---FSALDVLGSQNFSSGKYYWEVDVSKKIAWILGVYSKTSNLNRKKSSGIVFNPDVN
E. fuscus (XM_028137641.1)        QSYWVNLRLNPLNMLSNIVISEDRRQVTVGSYFMFGNVYPCD---FSSFDVLGSQNFSSGKYYWEVDVSKKIAWILGVYSKTSNLNRKKSSGFVFNPYAN
E. fuscus (XM_008160443.2)        QSYWVDLTLNPSNDLSNVVVSEDQRQVTVGNDFMFRNVYPCN---LSDFDVQGSQNFSSRKYYWEVDVSGKIAWVLGVYSKTSNLNRKKSSGFVFNRNPN
D. rotundus (XM_024574672.1)      QSYWVDLMLNPLNNLSNVAVSSDQRQVTFGQYSMFSNVYPCN---FSAFDVLGSRNFFSGKYYWEVDVSGKIAWILGVYSTPSNFNRKKSSGFVFNPNVN
D. rotundus (XM_024574678.1)      QSYWVDLMLNPLNNLSNVVISADQRQVTVGQHSVFSNVCPCNPCNFSAFDVVGSQHFSSGKCYWEVDVSGKIAWILGVYSTPSNFDRKKSSGFVFNPNVN
M. natalensis (XM_016214980.1)    QSYWVDLTMNPLSALSNVVISADQKQVTMGDDFMFRNVYPCN---FSALDVLGSKNFSSGKYYWEVDVSGKTAWILGVHSNTSNLNRKSSSGFVFNPNVN
P. alecto (XM_006915675.3)        QSYWVDIMLNPENSLSNVVISMDQRQVKAGDCFMFRNLHSCN---FSDFDAFGCQNFSSGKYYWEVDVSGKTAWILGVCRKTNNHFGKNSSGFVFKSNVN
R. aegyptiacus (XM_016150064.2    QSYWVDIMLDPVNCLSNAVISADQRQVQAGCCSMLKNAYPCN---FSNLDVFGCQNFSSGKYYWEVDVSGKIAWILGVCRKANNYFGENSSGFVFSPSGN
P. kuhlii (XM_036428149.1)        QNYWVNLMLNPLNTLSNIVISEDRRQITVGNYFMFGNVYPRD---FSSLDVLGSQNFSSGKYYWEVDVSKKIAWILGVHSKTSNLNRKKRSRFVFNPYAN
P. kuhlii (XM_036428108.1)        QSHWVDLTLNPFNDLSNVVVSEDHRQVTIGSDFMFRNVYPRN---FFAFDVQSSQNFSSGKYYWEVDVSGKVAWILGVYSKTSNLNRNKSSGFVFTPKPN
P. discolor (XM_036030172.1)      QSYWVDLMLNPLDAVSNVVISADQRQVTVGQHSMSSNVYPCNPCNFSAFDVVGNQHFSSGKYYWEVDVSGKIAWILGVYSTASNLNRKKSSGFVFNPNVN
P. discolor (XM_028516503.2)      QSYWVDLMLNPLNNLSNVAVSSDQRQVTFGQYSMFSNVYPCN---FSAFDVLGSRNFFSGKYYWEVDVSGKIAWILGVYSTPSNLNRKKSSGFVFNPNVN
R. ferrumequinum (XM_033118908    QSYWVDFMLNPVNALSGVVVSEDHRQVTAGNSFMFRNVYPCN---FSAFDVLGCQNFSSGKYYWEVDVSRKTAWILGVYSDRSDLNRKRSSGFVFNPHVN
S. hondurensis (XM_037051564.1    QSYWVDLMLNPLNAVSNVVISADQRQVTVGQQSTFSDVHPCNPCDFSAFDVVGTQHFSSGKYYWEVDVSGKIAWILGVYSAPSNLNSKKSSGFVFNPSVN
S. hondurensis (XM_037051559.1    QSYWVDLMLNPLNNLSNVAVSSDQRQVTFGQYSIFSNIYPCN---FSAFDVLGSRNFFSGKYYWEVDVSGKIAWILGVYSAPSNFNRKKSSGFVFNPNVN
M. molossus (XM_036259233.1)      QSHWVDLMLNPLYALSNVVVSADKRQVTVGNRFMFQSAYPCN---FSAFDVLGNQNFSSGKYYWEVDVSGKIAWVLGVYSNTSILNSKKSSGFFLNPNVN

                                          410       420       430       440       450       460       470       480       490       500
                                  ....|....|....|....|....|....|....|....|....|....|....|....|....|....|....|....|....|....|....|....|
H. armiger (XM_019668426.1)       Y-------QNV--YYRFRPENGYWVVGLQNESEYNAFEDSSTADPKVLTLSMAVVPCRVGVFLDYEAGTVSFFNVTNHGSLIYKFSKCRFSQTVYPYFNP
H. armiger (XM_019668416.1)       Y-------QNI--YSRFRPENGYWVVGLQNESEYNAFEDSSTADPKVLTLFMAVPPCRVGVFLDYEAGTLSFFNITNHGSLIYKFSKCRFPPTVYPYFNP
A. jamaicensis (XM_037139108.1    Y-------SND--YSRFRPENGYWVIGLQNKSEYSAFEDSPTSDPKVLTLYMAVPPRRVGVFLDFEAGAVSFLNITNQGSLIYKFSKCHFSQTAYPYFNP
A. jamaicensis (XM_037146302.1    D-------SNA--YSRFRPENGYWVIGLQNKSEYSAFEDSPTSDPKVLTLYMAVPPLRVGVFLDYEEGDVSFFNVTNQGSLIYKFSKCHFSQTAYPYFNP
M. brandtii (XM_014540011.1)      Y-------LNA--YSRFRPENGYWVVGLQNESEYNAFEDSSTSDPKVLTLSMAVAPCRVGVFLDYEANTVSFFNVTNHGSLIYKFSKCHFSQSVYPYFNP
M. brandtii (XM_014535246.1)      Y-------SNA--YSRFRPENGFWVVGLQNESEYNAFEDSSTSDPKVLTLSMFVPPRRVGVFLDYEASTVSFFNVTNHGSLIYKFSKCHFSHTVYPYFNP
M. lucifugus (XM_014460759.2)     Y-------LNA--YSRFRPENGYWVVGLQNESEYNAFEDSSTSDPKVLTLSMFVPPCRVGVFLDYEANTVSFFNVTNHGSLIYKFSKCHFSQTVYPYFNP
M. lucifugus (XM_023758783.1)     Y-------PNA--YSRFRPENGYWVVGLQNECEYNAFEDSSTSDPKVLTLSVFVPPHRVGVFLDYEASTVSFFNVTNHGSLIYKFSKCHFSHTVYPYFNP
M. myotis (XM_036325201.1)        Y-------PNA--YSRFRPENGYWVVGLQNASEYNAFEDSSTSDPKVLTLSMAVPPHRVGVFLDYEARTVSFFNVTNHGSLIYKFSKCHFSQTVYPYFNP
M. myotis (XM_036325210.1)        Y-------LNA--YSRFRPENGYWVVGLQNESEYNAFEDSSTSDPKVLTLSMFVPPCRVGVFLDYEARTVSFFNVTNHGSLIYKFSKCHFSQTVYPYFNP
M. davidii (XM_006770069.2)       Y-------LNA--YSRFRPENGYWVVGLQNESEYNAFEDSSTSDPKVLTLSMFVPPCRVGVFLDYEASTVSFFNVTNHGSLIYKFSKCHFSQVVYPYFNP
E. fuscus (XM_028137641.1)        Y-------PNA--YTRFRPENGYWVVGLQNESEYNAFEDSSTSDPKVFTLFMAVAPRRIGVFLDYEARTVSFFNVTNHGSLIYKFSKCHFSQTVYPYFNP
E. fuscus (XM_008160443.2)        Y-------SNA--YSRFRPENGYWVVGLQNESEYNAFEDSSTSDPKVLTLSMAVAPHRIGVFLDYEARTVSFFNVTNNGSLIYKFSKCHFSQTVYPYFNP
D. rotundus (XM_024574672.1)      H-------SNA--YSRFRPENGYWVIGLQDESEYRAF--TPTSDPKVLTLYMAAPPRRVGVFLDFEAGTVSFFNVTNQGSLIYKFSKCHFSQTAYPYFNP
D. rotundus (XM_024574678.1)      H-------SNA--YSRFRPENGYWVIGLQNEFEYSAFEDSPTSDPKVLTLYMAAPPRRVGVFLDFEAGTVSFFNVTNQGSLIYKFSKCHFSQTAYPYFNP
M. natalensis (XM_016214980.1)    Y-------PNV--YSRFKPENGYWVVGLHNESEYSAFEDSSTADPRVLTLSMAAPPRRVGVFLDYEAGTVSFFNVTNHGSLIYKFSKCCFSHAVYPYFNP
P. alecto (XM_006915675.3)        HYSKLRFDPNVNHYSKYRPENGYWVVGLRNISEYMAFEDASTSHPKVLTLFTAVPPRRVGVFLDYEAGIVSFFNITNHGSLIYKFSRCQFSQTAYPYFNP
R. aegyptiacus (XM_016150064.2    NYSEFFDDPNVDHYSKYRPQNGYWVVGLRDKSEYIAFEDASTSHPKILTLFMAVPPCRVGVFLDYEAGTVSFFNVTNHGSLIYRFSKCQFSQTAFPYFNP
P. kuhlii (XM_036428149.1)        Y-------QNA--YTRFRPENGYWVVGLQNESEYNAFEDSATSDPKVLTLPMAVAPHRIGVFLDYEASTVSFFNVTNHGSLIYKFSKCHFSQTVYPYFNP
P. kuhlii (XM_036428108.1)        Y-------SNA--YSRFRPENGYWVVGLQNQSEYNAFEDSSTTDPNILTLFIAVPPHRVGIFLDQEASTVTFFNVTNHGSLIYKFSKCHFSQTVYPYFNP
P. discolor (XM_036030172.1)      D-------SNA--YSRFRPENGFWVIGLQNESEYTAFEDSPTSDPKVLTLYMAVPPHRVGVFLDYEAGTVSFFNVTNQGSLIYKFSKCQFSQTAYPYFNP
P. discolor (XM_028516503.2)      Y-------SNA--YSRFRPENGFWVIGLQNESEYTAFEDSPTSDPKVLTLYMAVPPHRVGVFLDFEAGTVSFFNVTNQGSLIYKFSKCHFSQTAYPYFNP
R. ferrumequinum (XM_033118908    Y-------QTV--YSRFRPDNGYWVVGLQNVSEYNAFEDSSTSDPKVLTLSMAVPPCRVGVFLDYEAGTVSFFNVTNHRSLIYKFSKCRFSQAAYPYFNP
S. hondurensis (XM_037051564.1    D-------SNA--YSRFRPENGYWVIGLQNRSVYSAFQDSPTSDPKVLTLYMTVPPCRVGVFLDFEAGAVSFFNITNQGSLIYKFSNCHFSQTAYPYFNP
S. hondurensis (XM_037051559.1    Y-------SNA--YSKFRPENGYWVIGLQNESEYSAFEDSPTSDPKVLTFYMAVPPCRVGVFLDFEGGAVSFFNITNQGSLIYKFSNCHFSQTAYPYFNP
M. molossus (XM_036259233.1)      Y-------LNV--YSRFRPANGYWVVGLQNGCEYSAFEDSSTSDPKVLTLSMAVPPCRVGVFLDYEAGTVSFFNVTNHGSIIYKFSKCRFSQTAYPYFNP

                                          510
                                  ....|....|....
H. armiger (XM_019668426.1)       WHCPAPMTLCQPNS
H. armiger (XM_019668416.1)       WNCPAPMTLCQPNS
```

```
A. jamaicensis (XM_037139108.1  WNCPVPMTLCPPGS
A. jamaicensis (XM_037146302.1  WNCPVPMTLCPPGS
M. brandtii (XM_014540011.1)    WNCPAPMTLCPPSS
M. brandtii (XM_014535246.1)    WNCPVPMKLCPPSS
M. lucifugus (XM_014460759.2)   WNCPAPMILCPPSS
M. lucifugus (XM_023758783.1)   GNCPVPMKLCPPSS
M. myotis (XM_036325201.1)      WNCPVPMKLCPPSS
M. myotis (XM_036325210.1)      WNCPAPMTLCPPSS
M. davidii (XM_006770069.2)     WNCPAPMTLSPPSS
E. fuscus (XM_028137641.1)      CNCPAPLTLCPPSS
E. fuscus (XM_008160443.2)      WNCPVPMTLCPPSS
D. rotundus (XM_024574672.1)    WNCPVPMTLCPPGS
D. rotundus (XM_024574678.1)    WNCPVPMTLCPPGS
M. natalensis (XM_016214980.1)  WNCPVPMTLCPPSS
P. alecto (XM_006915675.3)      WNCLAPMTLCQPNS
R. aegyptiacus (XM_016150064.2  GNCPTPMTLCQPNS
P. kuhlii (XM_036428149.1)      CKCPVPMTLCPPSS
P. kuhlii (XM_036428108.1)      WNCPVPITLCPPSS
P. discolor (XM_036030172.1)    WNCPVPMTLCPPGS
P. discolor (XM_028516503.2)    WNCPVPMTLCPPGS
R. ferrumequinum (XM_033118908  WNCPTPMTLCQPNS
S. hondurensis (XM_037051564.1  WNCPVPMTLCPPGP
S. hondurensis (XM_037051559.1  WNCPVPMTLCPPGF
M. molossus (XM_036259233.1)    WNCPAPMTLCPPSS
```
